# Supplementary material for: Liver blood marker testing in UK primary care: a UK wide cohort study, 2004–2016
Source: BMJ Open. 2022 Sep 26;12(9):e058967. doi: 10.1136/bmjopen-2021-058967 (PMC9516205; doi:10.1136/bmjopen-2021-058967)

Supplementary Table S1 – Codes

**Liver blood tests** and their values were identified from the CPRD “test” dataset using the following entity file codes: ALT = 155; AST = 156; ALP = 153; GGT = 172; Bilirubin = 158; Platelet count = 189.			
**Type 2 diabetes**			
C100112	Non-insulin dependent diabetes mellitus	C109200	Non-insulin-dependent diabetes mellitus with neuro comps
C107400	NIDDM with peripheral circulatory disorder	C109211	Type II diabetes mellitus with neurological complications
C109.00	Non-insulin dependent diabetes mellitus	C109212	Type 2 diabetes mellitus with neurological complications
C109.11	NIDDM - Non-insulin dependent diabetes mellitus	C109300	Non-insulin-dependent diabetes mellitus with multiple comps
C109.12	Type 2 diabetes mellitus	C109312	Type 2 diabetes mellitus with multiple complications
C109.13	Type II diabetes mellitus	C109400	Non-insulin dependent diabetes mellitus with ulcer
C109000	Non-insulin-dependent diabetes mellitus with renal comps	C109411	Type II diabetes mellitus with ulcer
C109011	Type II diabetes mellitus with renal complications	C109412	Type 2 diabetes mellitus with ulcer
C109012	Type 2 diabetes mellitus with renal complications	C109500	Non-insulin dependent diabetes mellitus with gangrene
C109100	Non-insulin-dependent diabetes mellitus with ophthalm comps	C109511	Type II diabetes mellitus with gangrene
C109111	Type II diabetes mellitus with ophthalmic complications	C109512	Type 2 diabetes mellitus with gangrene
C109112	Type 2 diabetes mellitus with ophthalmic complications	C109600	Non-insulin-dependent diabetes mellitus with retinopathy
**Obesity** = BMI>30. Values obtained from entity file: weight =13 and height = 14. Calculate BMI for every available weight measurement (using single height measurement). If BMI > 30 at any stage up to end of follow-up for individual patient then include in Obesity population. Index date is first occurrence of BMI >30.			
**Cirrhosis:** Identified from READ codes/terms			
7609	Open operations on oesophageal varices	J612.12	Laennec's cirrhosis
7609300	Local ligation of oesophageal varices	J615.00	Cirrhosis - non alcoholic
7609400	Open injection sclerotherapy to oesophageal varices	J615.11	Portal cirrhosis
7609y11	Tanner devascularisation for bleeding varices	J615100	Multilobular portal cirrhosis
7609z00	Open operation on oesophageal varices NOS	J615300	Diffuse nodular cirrhosis
760C300	Fibreoptic endoscopic injection sclerotherapy oesoph varices	J615400	Fatty portal cirrhosis
760C500	Fibreoptic endoscopic banding of oesophageal varices	J615500	Hypertrophic portal cirrhosis
760F300	Rigid oesophagoscopic injection sclerotherapy oesoph varices	J615600	Capsular portal cirrhosis
760F400	Rigid oesophagoscopic banding of oesophageal varices	J615700	Cardiac portal cirrhosis
761D800	Fibreopt endoscop rubber band ligation of upper GIT varices	J615800	Juvenile portal cirrhosis
C310400	Glycogenosis with hepatic cirrhosis	J615812	Indian childhood cirrhosis

C350012	Pigmentary cirrhosis of liver	J615C00	Xanthomatous portal cirrhosis
G85..11	Oesophageal varices	J615D00	Bacterial portal cirrhosis
G850.00	Oesophageal varices with bleeding	J615H00	Infectious cirrhosis NOS
G851.00	Oesophageal varices without bleeding	J615y00	Portal cirrhosis unspecified
G852.00	Oesophageal varices in diseases EC	J615z00	Non-alcoholic cirrhosis NOS
G852000	Oesophageal varices with bleeding in diseases EC	J615z11	Macronodular cirrhosis of liver
G852100	Oesophageal varices without bleeding in diseases EC	J615z12	Cryptogenic cirrhosis of liver
G852200	Oesophageal varices in cirrhosis of the liver	J615z13	Cirrhosis of liver NOS
G852300	Oesophageal varices in alcoholic cirrhosis of the liver	J616.00	Biliary cirrhosis
G852z00	Oesophageal varices in diseases EC NOS	J616100	Secondary biliary cirrhosis
G857.00	Gastric varices	J616200	Biliary cirrhosis of children
G858.00	Oesophageal varices NOS	J616z00	Biliary cirrhosis NOS
J61..00	Cirrhosis and chronic liver disease	J623.00	Portal hypertension
J612.00	Alcoholic cirrhosis of liver	J635600	Toxic liver disease with fibrosis and cirrhosis of liver
J612.11	Florid cirrhosis	Jyu7100	[X]Other and unspecified cirrhosis of liver
**Alcohol excess:** Identified from units per week from entity file (entity=5) and READ codes/terms			
136K.00	Alcohol intake above recommended sensible limits	E231.00	Chronic alcoholism
136S.00	Hazardous alcohol use	E231z00	Chronic alcoholism NOS
136T.00	Harmful alcohol use	E23z.00	Alcohol dependence syndrome NOS
136W.00	Alcohol misuse	E250.00	Nondependent alcohol abuse
8H7p.00	Referral to community alcohol team	E250000	Nondependent alcohol abuse, unspecified
9NN2.00	Under care of community alcohol team	E250200	Nondependent alcohol abuse, episodic
9k1..00	Alcohol misuse - enhanced services administration	E250z00	Nondependent alcohol abuse NOS
9k1A.00	Brief intervention for excessive alcohol consumption completed	Eu10211	[X]Alcohol addiction
E23..00	Alcohol dependence syndrome	Eu10212	[X]Chronic alcoholism
E23..11	Alcoholism	ZV11300	[V]Personal history of alcoholism
E23..12	Alcohol problem drinking		

Supplementary Table S2 – Total numbers of tests measured and individuals being tested

	2004	2005	2006	2007	2008	2009	2010	2011	2012	2013	2014	2015	2016
**ALT**													
Total tests	145,762	182,250	220,863	264,573	299,956	336,059	349,857	355,185	377,633	379,820	336,022	274,827	199,196
Total people tested	101,485	126,562	152,516	183,196	206,761	228,380	239,920	246,920	261,544	264,206	233,037	193,654	141,885
**AST**													
Total tests	62,414	69689	72,998	69218	75,502	81218	78,629	79997	71,305	53,714	44,064	36,542	18,363
Total people tested	44,294	49158	51,781	48791	53,406	56756	56,085	57243	50,856	39,178	31,411	26,275	13,075
**ALP**													
Total tests	199,350	241693	288,224	326253	368,076	411994	426,350	442311	461,329	454,319	405,753	332,655	236,886
Total people tested	136,121	163894	192,150	218956	245,608	269713	280,957	292771	302,822	296,931	261,378	215,265	156,549
**GGT**													
Total tests	78,572	92452	102,634	111127	119,522	127386	132,905	127210	116,707	102,085	74,190	61,516	40,953
Total people tested	54,346	64692	71,192	78118	84,354	88723	93,147	90618	82,859	75,125	55,022	45,726	30,507
**Bilirubin**													
Total tests	192,632	231683	275,631	311066	350,373	388612	399,189	411125	426,237	412,887	366,861	299,687	215,528
Total people tested	134,046	160842	189,233	215296	241,713	264757	274,782	286590	295,722	288,212	254,867	210,081	152,916
**Platelet count**													
Total tests	269,019	318699	368,616	419187	475,732	524655	540,431	555732	589,239	557,555	495,158	389,745	279,714
Total people tested	158,987	185336	211,828	238400	265,257	289243	300,868	314311	324,861	315,432	279,168	230,590	167,506

Supplementary Table S3 Prevalence of liver blood markers over time

	2004	2005	2006	2007	2008	2009	2010	2011	2012	2013	2014	2015	2016
**ALT**													
All	13.0%<br>101,485	14.7%<br>126,562	16.1%<br>152,516	17.8%<br>183,196	19.0%<br>206,761	20.0%<br>228,380	20.5%<br>239,920	20.9%<br>246,920	22.0%<br>261,544	23.2%<br>264,206	23.0%<br>233,037	23.1%<br>193,654	23.7%<br>141,885
No risk factor	10.2%<br>58,099	11.5%<br>71,085	12.3%<br>82,630	13.7%<br>97,925	14.6%<br>109,206	15.4%<br>119,415	15.6%<br>122,910	15.9%<br>125,077	16.7%<br>131,327	17.5%<br>131,491	17.4%<br>115,211	17.3%<br>93,323	17.9%<br>69,323
Alcohol excess	15.3%<br>15,571	17.2%<br>19,838	19.2%<br>24,616	21.5%<br>30,304	23.1%<br>34,840	24.4%<br>38,992	25.1%<br>41,558	25.6%<br>42,907	27.1%<br>45,955	28.3%<br>46,207	28.0%<br>41,346	27.4%<br>34,669	28.3%<br>23,270
Obesity	21.9%<br>25,460	24.6%<br>33,357	27.2%<br>42,787	29.2%<br>53,140	30.6%<br>61,862	31.6%<br>70,159	32.4%<br>76,530	32.5%<br>80,517	33.9%<br>86,275	35.4%<br>88,493	35.0%<br>79,136	35.1%<br>68,224	35.7%<br>50,029
Type 2 diabetes	56.1%<br>10,271	61.0%<br>13,741	64.4%<br>17,680	67.9%<br>21,964	68.4%<br>24,670	68.1%<br>27,140	68.9%<br>29,564	67.4%<br>31,100	68.5%<br>33,335	70.7%<br>34,909	68.4%<br>31,408	68.6%<br>27,066	68.8%<br>20,317
All 3 risk factors	59.1%<br>874	63.9%<br>1247	68.4%<br>1685	71.7%<br>2186	72.4%<br>2613	71.9%<br>3035	71.5%<br>3430	70.7%<br>3769	70.3%<br>3983	73.0%<br>4260	71.3%<br>3931	70.2%<br>3510	71.1%<br>2436
**AST**													
All	5.7%<br>44,294	5.7%<br>49,158	5.5%<br>51,781	4.7%<br>48,791	4.9%<br>53,406	5.0%<br>56,756	4.8%<br>56,085	4.8%<br>57,243	4.3%<br>50,856	3.4%<br>39,178	3.1%<br>31,411	3.1%<br>26,275	2.2%<br>13,075
No risk factor	4.4%<br>25,237	4.5%<br>27,674	4.1%<br>27,556	3.6%<br>25,603	3.7%<br>27,614	3.7%<br>28,920	3.6%<br>28,504	3.7%<br>29,130	3.3%<br>25,902	2.6%<br>19,882	2.3%<br>15,406	2.3%<br>12,526	1.6%<br>6,017
Alcohol excess	6.5%<br>6,672	6.5%<br>7,446	6.5%<br>8,311	5.5%<br>7,708	5.6%<br>8,497	5.7%<br>9,129	5.5%<br>9,123	5.5%<br>9,214	4.8%<br>8,206	3.9%<br>6,298	3.6%<br>5,368	3.8%<br>4,791	3.0%<br>2,503
Obesity	9.8%<br>11,411	9.7%<br>13,137	9.6%<br>15,047	8.2%<br>14,942	8.4%<br>17,073	8.3%<br>18,511	7.8%<br>18,354	7.6%<br>18,929	6.6%<br>16,709	5.3%<br>13,124	4.8%<br>10,921	4.8%<br>9,327	3.4%<br>4,740
Type 2 diabetes	24.3%<br>4,444	23.6%<br>5,309	22.8%<br>6,257	18.9%<br>6,104	18.9%<br>6,808	18.4%<br>7,318	16.8%<br>7,228	16.0%<br>7,387	13.6%<br>6,599	10.3%<br>5,099	9.1%<br>4,155	9.6%<br>3,780	6.5%<br>1,919
All 3 risk factors	24.9%<br>369	24.4%<br>477	22.6%<br>557	18.9%<br>577	18.7%<br>676	18.0%<br>759	16.5%<br>793	15.3%<br>813	12.7%<br>721	9.9%<br>576	9.2%<br>506	9.6%<br>478	7.4%<br>252
**ALP**													

All	17.4%	19.0%	20.3%	21.3%	22.6%	23.7%	24.0%	24.8%	25.5%	26.0%	25.8%	25.6%	26.2%
	136,121	163,894	192,150	218,956	245,608	269,713	280,957	292,771	302,822	296,931	261,378	215,265	156,549
No risk	13.8%	14.9%	15.6%	16.4%	17.4%	18.2%	18.3%	19.0%	19.5%	19.9%	19.7%	19.4%	19.9%
factor	78,382	92,690	104,627	117,407	129,995	141,235	144,684	149,282	153,272	149,168	130,327	104,727	76,926
Alcohol	20.0%	21.7%	23.8%	25.0%	26.6%	27.9%	28.6%	29.4%	30.5%	31.0%	30.7%	29.9%	31.1%
excess	20,355	25,039	30,447	35,275	40,123	44,632	47,212	49,230	51,624	50,602	45,304	37,836	25,565
Obesity	29.5%	31.9%	34.3%	35.1%	36.8%	37.7%	38.1%	38.7%	39.2%	39.7%	39.1%	38.8%	39.2%
	34,314	43,222	53,982	63,964	74,331	83,756	89,904	95,755	99,898	99,094	88,395	75,373	54,922
Type 2	73.5%	77.4%	79.6%	80.4%	81.0%	80.6%	80.2%	79.3%	78.6%	78.2%	75.5%	75.1%	75.0%
diabetes	13,458	17,429	21,845	26,018	29,210	32,115	34,420	36,568	38,245	38,590	34,647	29,619	22,161
All 3 risk	75.0%	79.1%	82.1%	82.0%	82.7%	81.9%	80.8%	80.8%	78.9%	78.7%	77.1%	75.5%	77.2%
factors	1,110	1,544	2,021	2,501	2,986	3,455	3,876	4,305	4,471	4,588	4,251	3,774	2,645
**GGT**													
All	7.0%	7.5%	7.5%	7.6%	7.8%	7.8%	8.0%	7.7%	7.0%	6.6%	5.4%	5.4%	5.1%
	54,346	64,692	71,192	78,118	84,354	88,723	93,147	90,618	82,859	75,125	55,022	45,726	30,507
No risk	5.3%	5.6%	5.5%	5.5%	5.6%	5.6%	5.7%	5.5%	5.0%	4.7%	3.9%	3.9%	3.7%
factor	29,868	34,865	36,661	39,595	42,214	43,632	45,103	43,616	39,378	35,625	25,434	20,846	14,408
Alcohol	9.7%	10.3%	10.8%	11.0%	11.3%	11.5%	11.7%	11.2%	10.4%	9.6%	8.3%	7.9%	7.3%
excess	9,837	11,909	13,845	15,572	17,021	18,307	19,323	18,790	17,571	15,738	12,271	9,922	5,971
Obesity	11.8%	12.8%	12.9%	12.6%	12.8%	12.6%	12.8%	12.1%	10.9%	10.2%	8.4%	8.5%	7.7%
	13,754	17,296	20,350	23,024	25,877	27,954	30,300	29,929	27,680	25,385	19,030	16,469	10,731
Type 2	28.6%	29.3%	28.0%	27.1%	26.2%	25.1%	25.3%	23.7%	20.7%	19.2%	14.8%	15.1%	13.1%
diabetes	5,232	6,601	7,693	8,757	9,456	10,007	10,855	10,946	10,061	9,484	6,796	5,942	3,879
All 3 risk	32.2%	33.8%	33.0%	32.2%	31.3%	31.1%	30.1%	29.4%	24.6%	22.6%	18.3%	17.8%	14.8%
factors	476	659	812	982	1,131	1,314	1,443	1,568	1,394	1,319	1,009	891	507
**Bilirubin**													
All	17.2%	18.6%	20.0%	20.9%	22.2%	23.2%	23.5%	24.3%	24.9%	25.3%	25.2%	25.0%	25.6%
	134,046	160,842	189,233	215,296	241,713	264,757	274,782	286,590	295,722	288,212	254,867	210,081	152,916
	13.5%	14.6%	15.3%	16.1%	17.0%	17.8%	17.8%	18.4%	18.9%	19.2%	19.1%	18.8%	19.3%

No risk factor	76,925	90,589	102,695	114,978	127,452	137,983	140,694	145,318	148,912	143,914	126,200	101,520	74,600
Alcohol excess	19.8%	21.5%	23.5%	24.7%	26.4%	27.6%	28.2%	29.0%	30.0%	30.3%	30.2%	29.4%	30.7%
	20,174	24,766	30,144	34,890	39,753	44,116	46,549	48,609	50,790	49,536	44,537	37,217	25,195
Obesity	29.1%	31.4%	33.9%	34.7%	36.3%	37.2%	37.4%	38.0%	38.5%	38.7%	38.3%	38.0%	38.5%
	33,896	42,574	53,306	63,125	73,377	82,584	88,397	94,097	97,916	96,601	86,706	73,942	53,945
Type 2 diabetes	73.2%	76.9%	79.1%	79.9%	80.5%	80.0%	79.5%	78.7%	77.7%	77.0%	74.8%	74.5%	74.4%
	13,402	17,306	21,709	25,850	29,030	31,878	34,108	36,294	37,812	38,009	34,346	29,378	21,978
All 3 risk factors	74.8%	78.7%	81.6%	81.8%	82.4%	81.6%	80.5%	80.4%	78.2%	77.9%	76.6%	75.0%	76.8%
	1,107	1,536	2,010	2,494	2,976	3,443	3,862	4,284	4,430	4,544	4,222	3,749	2,629
**Platelets**													
All	20.3%	21.5%	22.4%	23.2%	24.4%	25.4%	25.7%	26.6%	27.4%	27.6%	27.6%	27.5%	28.0%
	158,987	185,336	211,828	238,400	265,257	289,243	300,868	314,311	324,861	315,432	279,168	230,590	167,506
No risk factor	18.3%	19.1%	19.5%	20.1%	21.0%	21.8%	21.8%	22.5%	23.0%	23.1%	22.9%	22.4%	22.9%
	104,008	118,472	130,674	144,262	157,524	168,847	172,317	177,447	180,888	173,265	151,378	121,000	88,638
Alcohol excess	18.6%	19.9%	21.4%	22.5%	24.0%	25.2%	26.0%	27.0%	28.4%	29.0%	29.1%	29.0%	30.0%
	18,994	22,933	27,424	31,684	36,207	40,327	42,991	45,170	48,143	47,396	42,957	36,654	24,657
Obesity	28.7%	30.6%	32.4%	33.1%	34.7%	35.9%	36.5%	37.5%	38.3%	38.6%	38.6%	38.9%	39.4%
	33,393	41,452	50,954	60,244	70,172	79,767	86,087	92,765	97,383	96,496	87,227	75,562	55,216
Type 2 diabetes	47.9%	51.1%	54.4%	56.1%	58.3%	59.5%	61.9%	63.2%	64.0%	64.4%	63.7%	65.3%	66.3%
	8,764	11,511	14,932	18,153	21,031	23,689	26,553	29,146	31,107	31,793	29,233	25,752	19,593
All 3 risk factors	43.9%	48.6%	51.9%	52.9%	56.0%	56.9%	59.1%	61.2%	61.4%	63.0%	62.7%	64.2%	67.8%
	650	949	1,278	1,614	2,020	2,403	2,835	3,261	3,477	3,674	3,455	3,210	2,323

Supplementary Figure S1 Prevalence of liver marker testing over time by the subgroups: no liver risk factors, Type 2 diabetes, excess alcohol consumption and obesity

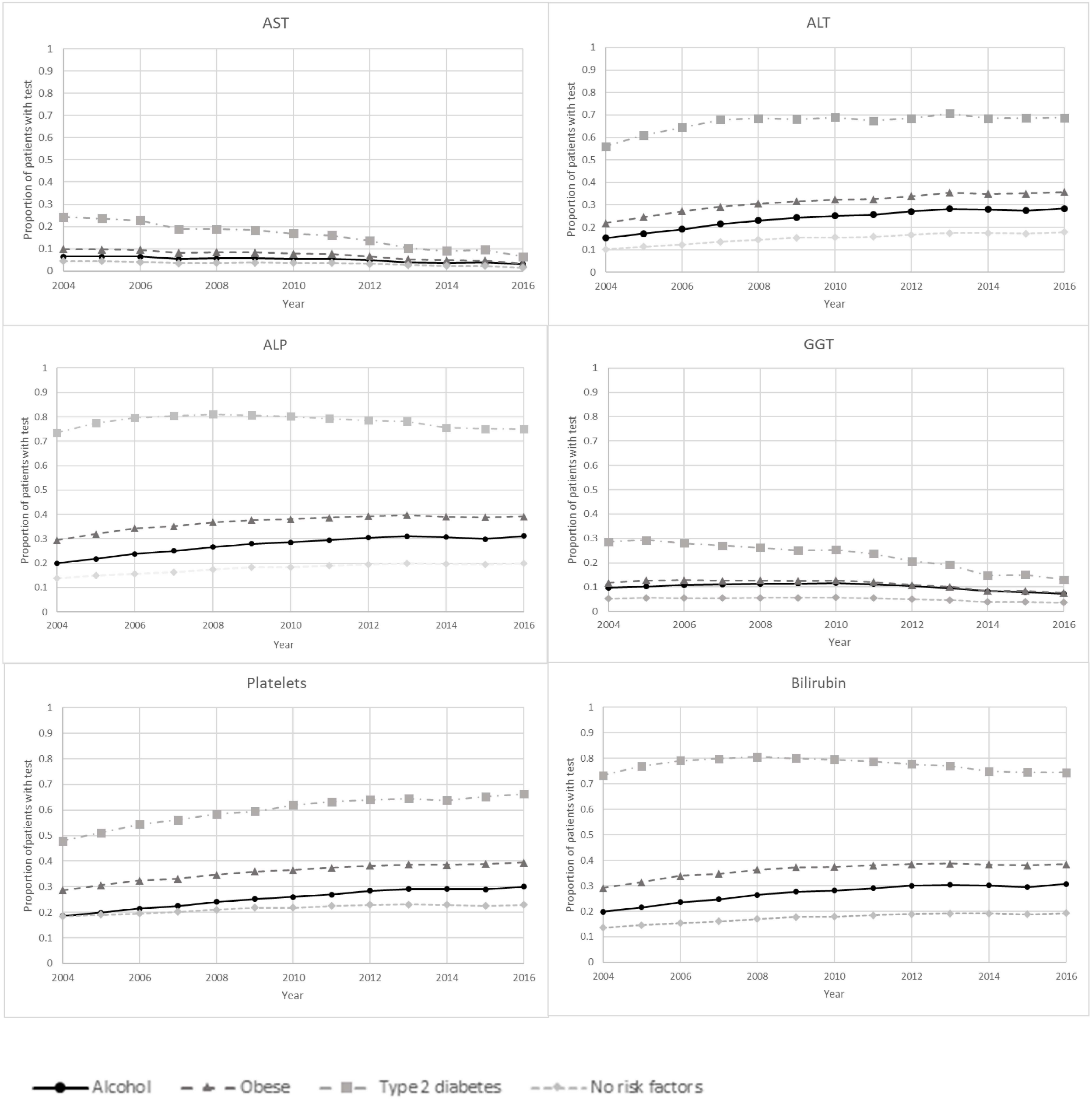

Supplement: Supplementary data [file bmjopen-2021-058967supp001.pdf]
