# Supplementary material for: Liver blood marker testing in UK primary care: a UK wide cohort study, 2004–2016
Source: BMJ Open. 2022 Sep 26;12(9):e058967. doi: 10.1136/bmjopen-2021-058967 (PMC9516205; doi:10.1136/bmjopen-2021-058967)
Supplement: Author's manuscript [file bmjopen-2021-058967.draft_revisions.pdf]

BMJ Open is committed to open peer review. As part of this commitment we make the peer review history of every article we publish publicly available.

When an article is published we post the peer reviewers' comments and the authors' responses online. We also post the versions of the paper that were used during peer review. These are the versions that the peer review comments apply to.

The versions of the paper that follow are the versions that were submitted during the peer review process. They are not the versions of record or the final published versions. They should not be cited or distributed as the published version of this manuscript.

BMJ Open is an open access journal and the full, final, typeset and author-corrected version of record of the manuscript is available on our site with no access controls, subscription charges or pay-per-view fees (http://bmjopen.bmj.com).

If you have any questions on BMJ Open's open peer review process please email
info.bmjopen@bmj.com

BMJ Open

Liver blood marker testing in UK primary care: a UK wide cohort study, 2004-2016

Journal:	*BMJ Open*
Manuscript ID	bmjopen-2021-058967
Article Type:	Original research
Date Submitted by the Author:	04-Nov-2021
Complete List of Authors:	Scutt, Polly; University of Nottingham, Stroke, Division of Clinical Neuroscience<br>Ban, Lu; University of Nottingham<br>Card, Tim; University of Nottingham<br>Crooks, Colin; University of Nottingham, Epidmiology and Public Health<br>Guha, Neil; University of Nottingham<br>West, Joe; University of Nottingham<br>Morling, Joanne; University of Nottingham
Keywords:	Adult gastroenterology < GASTROENTEROLOGY, Health policy < HEALTH SERVICES ADMINISTRATION & MANAGEMENT, PREVENTIVE MEDICINE, PRIMARY CARE

SCHOLARONE™
Manuscripts

I, the Submitting Author has the right to grant and does grant on behalf of all authors of the Work (as defined in the below author licence), an exclusive licence and/or a non-exclusive licence for contributions from authors who are: i) UK Crown employees; ii) where BMJ has agreed a CC-BY licence shall apply, and/or iii) in accordance with the terms applicable for US Federal Government officers or employees acting as part of their official duties; on a worldwide, perpetual, irrevocable, royalty-free basis to BMJ Publishing Group Ltd ("BMJ") its licensees and where the relevant Journal is co-owned by BMJ to the co-owners of the Journal, to publish the Work in this journal and any other BMJ products and to exploit all rights, as set out in our licence.

The Submitting Author accepts and understands that any supply made under these terms is made by BMJ to the Submitting Author unless you are acting as an employee on behalf of your employer or a postgraduate student of an affiliated institution which is paying any applicable article publishing charge ("APC") for Open Access articles. Where the Submitting Author wishes to make the Work available on an Open Access basis (and intends to pay the relevant APC), the terms of reuse of such Open Access shall be governed by a Creative Commons licence – details of these licences and which Creative Commons licence will apply to this Work are set out in our licence referred to above.

Other than as permitted in any relevant BMJ Author's Self Archiving Policies, I confirm this Work has not been accepted for publication elsewhere, is not being considered for publication elsewhere and does not duplicate material already published. I confirm all authors consent to publication of this Work and authorise the granting of this licence.

For peer review only

Liver blood marker testing in UK primary care: a UK wide cohort study, 2004-2016

For peer review only

[Liver blood tests in UK primary care]

Scutt Polly[1], Ban Lu[1,2], Card Tim C[1,3], Crooks Colin[3,4], Guha I Neil[3,4], West Joe[1,3], Morling Joanne R[1,3]

1. Population and Lifespan Sciences, School of Medicine, University of Nottingham, Clinical Sciences Building 2, City Hospital, Nottingham, UK, NG5 1PB
2. Evidera by PPD
3. NIHR Nottingham Biomedical Research Centre (BRC), Nottingham University Hospitals NHS Trust and the University of Nottingham, Nottingham, UK, NG7 2UH
4. Nottingham Digestive Diseases Centre (NDDC), School of Medicine, University of Nottingham, Nottingham, UK, NG7 2UH

Corresponding author: Dr Jo Morling

Population and Lifespan Sciences, School of Medicine, University of Nottingham,

Clinical Sciences Building 2, City Hospital, Nottingham, UK, NG5 1PB Joanne.morling@nottingham.ac.uk

Words: 2,046

Keywords: screening, early diagnosis, aspartate aminotransferase, policy, population

Summary/Abstract

Objective

We aimed to determine i) the temporal trends of liver enzyme testing in UK general practice and ii) how these vary amongst different subgroups at risk of CLD.

Design

Retrospective cohort study

Setting

UK primary care database (Clinical Practice Research Datalink (CPRD)), 2004-2016

Participants:

Patients aged 18 years or over, registered in the CPRD from 1st January 2004 to 31st December 2016,

Outcome measures

The frequency of testing recorded within the study period in general practice was calculated for: Alanine aminotransferase (ALT); Aspartate aminotransferase (AST); Gamma glutamyl transferase (GTT); Alkaline phosphatase (ALP); Bilirubin and platelets. Analyses were conducted in subgroups of patients at high risk of developing liver disease.

Results

The study cohort included 2,912,066 individuals with median follow-up of 3.2 years. The proportion of patients with at least one measurement for ALT, ALP, Bilirubin or platelet test gradually increased over the course of the study period and fell for AST and GGT. By 2016 the proportion of the population receiving one of more tests in that year was: platelet count 28.0%, ALP 26.2%, bilirubin 25.6%, ALT 23.7%, GGT 5.1% and AST 2.2%. Those patients with risk factors for CLD had higher proportions receiving liver marker assessments than those without risk factors.

For peer review only

Conclusions

The striking finding that AST is now only measured in a fraction of the population has significant implications for policy and practice. A more nuanced approach where non-invasive markers are targeted towards individuals with risk factors for CLD may be a solution.

[word count = 246]

Article summary

Strengths and limitations of this study

- Sampling frame: a significant strength is the use of a large national dataset (>15mill people).
- Data quality: the dataset used (CPRD) known to be representative of the UK population in terms of age, gender and geographical location with robust quality controls.
- Data validity: previously validated code lists were used for the identification of subgroups.
- A key limitation is the lack of information on the indication for testing or the resultant actions which limits interpretation to some degree.
- Since this study only includes people who attend the GP, and some of the individuals at highest risk of CLD will not be attending. Therefore, we underestimate the proportions potentially identified is systematic testing was employed.

For peer review only

Introduction

In the UK liver disease is a significant and growing burden on the National Health Service (NHS) and is the United Kingdom's third most common cause of premature mortality[1]; between 2015 and 2017 it caused 26,265 premature deaths in England alone[2]. It is also a significant source of healthcare inequity, with the median age of death differing by 9 years between the most and least deprived quintiles[3]. There has been a 400% increase in liver disease mortality in the population as a whole since 1970 and nearly 500% increases in mortality observed in working age populations over in this period[4].

Three independent reports since 2014 have highlighted the need for the early detection of liver disease including the Chief Medical Officer report (2012)[5], the All-Party Parliamentary Hepatology Group Inquiry[6] and the Lancet commission[4], in order to allow intervention and change the course of the disease. A number of organizations have now developed guidance advocating the use of non-invasive fibrosis markers in risk stratification[7–9]. Despite this, many existing community diagnostic pathways for detection and onward referral of suspected CLD are based on traditional liver enzyme tests which lack accuracy and result in delays to diagnosis[10].

The optimal non-invasive fibrosis marker is yet to be determined, however there are simple algorithms involving easily accessible measures such as AST and platelets that can be conducted in primary care, e.g. aspartate to platelet ratio index (APRI)[11], Fibrosis-4 score (FIB4)[12], and CIRRUS[13]. However, there is little understanding about how liver enzymes are currently used in UK general practice in order to support the implementation of changing practice and policy.

Given the rising prevalence of lifestyle related CLD and growing knowledge of non-invasive fibrosis measures one could hypothesise that there should have been a shift away from liver enzyme testing over time (shifting to non-invasive assessment). The aim of this study was to determine i) the temporal trends of liver enzyme testing in UK general practice and ii) how these vary amongst different subgroups at risk of CLD.

For peer review only

Materials and Methods

Data source

A population-based cohort study was conducted using the Clinical Practice Research Datalink (CPRD). The CPRD contains primary care data on 15.5 million people from 734 practices in the UK and is considered representative of the UK population[14]. Data are anonymised at patient and practice level and contain information on patient demographics, consultations, diagnoses, referrals and prescriptions. Clinical information is entered using READ codes which was a standard clinical terminology system used in the UK. For a subset of English practices (58% of UK CPRD practices), primary care data can be linked with the Hospital Episode Statistics (HES) dataset containing information all hospital admissions[15,16]. The population for this study consists only of patients from these practices eligible for linkage with the HES dataset. This was a fully annonymised databased study not requiring ethical approval. This use of the data for this study was approved by the Independent Scientific Advisory Committee (ISAC) for MHRA database (protocol 19_256).

Study Population

Patients aged 18 years or over, registered in the CPRD from 1st January 2004 to 31st December 2016, and having at least one day of registration with a practice eligible for linkage with the HES dataset were eligible for inclusion in the study. Patients with a diagnosis of CLD before the start of their follow-up period were excluded from the population. Patients were followed up starting at the latest of either the day after the date of current registration with their GP practice, the start of the study period or the date the GP practice was labelled "up to standard" (UTS). Follow-up ended at the earliest of either the date of death, date the patient transferred out of the GP practice, last date of data collection for the GP practice the patient is registered with, the end of the study period or the date of diagnosis with CLD in primary care.

Outcomes

The frequency of testing recorded within the study period in general practice for the following liver blood tests was calculated: Alanine aminotransferase (ALT); Aspartate aminotransferase (AST); Gamma glutamyl transferase (GTT); Alkaline phosphatase (ALP); Bilirubin and platelet count. Abnormal results

For peer review only

for each test were defined as: ALT result >50 (IU/L); AST result >40 (IU/L); ALP result >130 (IU/L); GGT result >50 (IU/L); Bilirubin result >21 (IU/L); platelet result <150 (platelets/mcl).

Subgroups

Analyses were conducted in the following subgroups of patients at high risk of developing CLD: presence of type 2 diabetes defined using READ codes (see Supplementary Table S1); obesity defined as a BMI >30 calculated using height and weight measures; use of alcohol defined as alcohol abuse using READ codes (see supplementary material) or recorded >14 units per week alcohol consumption. For all subgroups, follow-up for an individual patient started at the date of diagnosis in primary care. Patients who were diagnosed with CLD within their follow up period had their follow-up shortened to end 3 months before their date of diagnosis with CLD. An analysis of the subgroup of patients not included in any of these high-risk subgroups was also performed.

Statistical Analysis

The frequency of liver enzyme testing was presented as the proportion of patients with one or more tests out of the total eligible population over the study period. The frequency of abnormal test results was calculated and presented as the proportion of non-missing test results with an abnormal value. The number of tests performed per year on an individual was calculated by dividing the number of tests performed in the individual's follow-up period divided by the total length of their follow-up period. The proportion of patients with an AST test within 6 weeks following an abnormal ALT test result was calculated.

All analyses were conducted overall and stratified by sex, age group (18 – 29, 30 – 39, 40 – 49, 50 – 59, 60 –69, 70 – 79, 80+ years) and calendar year. Analyses were performed on the whole study population and in the risk subgroups.

Analyses were performed using SAS version 9.4.

Patient and Public Involvement

This study involved members of the Nottingham Digestive Diseases Biomedical Research Unit Patient Advisory Group t the following stages: research design and funding application, lay dissemination and discussion of results.

For peer review only

Results

Characteristics

The study cohort included 2,912,066 individuals with follow-up during the years 2004 – 2016 (median follow-up 3.2 years, IQR 1.3-6.9). Of these, the predefined risk factor subgroups contained: 550,185 (18.9%) with obesity, 384,011 (13.2%) with excess alcohol use, 120,305 (4.1%) with type 2 diabetes and 2,235,938 (76.8%) with none of the three risk factors (some individuals had more than one risk factor).

The most frequently measured blood marker was platelet count, with 49% of patients having at least one platelet count measured during their follow-up period. The least commonly measured was the AST level with only 10% of patients having at least one measurement in their follow-up. For all tests, the prevalence of testing increased with increasing age, with the highest proportion of patients being tested in the 70 – 80 year age category. Markers were more frequently measured in women and this difference was statistically significant for all markers ($p<0.0001$). Full details are given in Table 1.

Of those participants having tests the median number of tests undertaken each year was 1, however some individuals had in excess of 100 of the same test per year. Platelet count was most likely to be tested more than once in an individual with the other liver markers being similar (for additional detail see Supplementary Table S2).

Prevalence of marker measurement over time

The proportion of patients in the study population with at least one measurement for ALT, ALP, Bilirubin or platelet test gradually increased over the course of the study period (2004 – 2016) but conversely fell for AST and GGT markers (Figure 1 and Table 2). By 2016 the proportion of the population receiving one of more tests in that year was: platelet count 28.0%, ALP 26.2%, bilirubin 25.6%, ALT 23.7%, GGT 5.1% and AST 2.2%.

Prevalence of abnormal measures

The proportion of all tests being measured as abnormal remained generally static over the study period (Figure 2). Of the 3,922,529 (total number) of ALT test, 343,474 (8.8%) had an abnormal value. The first abnormal ALT test for each patient (N= 160,191) was paired with an AST test measurement within 6 weeks for 13,997 (8.7%). The proportion of measurements with abnormal values for all other markers

For peer review only

was also low: AST (7.5%), ALP (7.9%), GGT (24.6%), Bilirubin (4.7%), platelets (16.0%) and these proportions remained stable over the study period.

For peer review only

Table 1 Characteristics of participants (ever measurements)

		Whole population (N)	ALT		AST		ALP		GGT		Bilirubin		Platelet count	
			1+ tests	1+ abnormal	1+ tests	1+ abnormal	1+ tests	1+ abnormal	1+ tests	1+ abnormal	1+ tests	1+ abnormal	1+ tests	1+ abnormal
All	n	2,912,066	1,112,879	160,191	284,274	33743	1,261,596	140,433	459,754	124,475	1,246,003	99,633	1,414,798	301,127
	%		38.2%	14.4%	9.8%	11.9%	43.3%	11.1%	15.8%	27.1%	42.8%	8.0%	48.6%	21.3%
Sex														
Male	n	1,378,945	484,471	102,962	125,241	19,852	547,936	57,766	213,668	76,266	542,549	62,838	555,508	124,432
	%		35.1%	21.3%	9.1%	15.9%	39.7%	10.5%	15.5%	35.7%	39.3%	11.6%	40.3%	22.4%
Female	n	1,533,121	628,408	57,229	159,033	13,891	713,660	82,667	246,086	48,209	703,454	36,795	859,290	176,695
	%		41.0%	9.1%	10.4%	8.7%	46.5%	11.6%	16.1%	19.6%	45.9%	5.2%	56.0%	20.6%
Age group, years														
18 – 29	n	1,117,738	196,244	19,026	46,304	3,311	230,084	15,495	70,498	7,038	225,208	15,343	328,696	65,280
	%		17.6%	9.7%	4.1%	7.2%	20.6%	6.7%	6.3%	10.0%	20.1%	6.8%	29.4%	19.9%
30 – 39	n	1,000,314	246,015	35,067	61,081	6,127	287,277	18,522	95,123	19,394	281,707	18,460	370,007	74,137
	%		24.6%	14.3%	6.1%	10.0%	28.7%	6.4%	9.5%	20.4%	28.2%	6.6%	37.0%	20.0%
40 – 49	n	718,585	266,922	42,817	66,307	7,793	307,249	19,276	108,280	30,790	303,132	20,614	324,775	62,005
	%		37.1%	16.0%	9.2%	11.8%	42.8%	6.3%	15.1%	28.4%	42.2%	6.8%	45.2%	19.1%
50 - 59	n	476,113	221,016	36,113	54,770	72,51	251,719	24,052	91,799	30,896	248,874	16,947	247,761	47,522
	%		46.4%	16.3%	11.5%	13.2%	52.9%	9.6%	19.3%	33.7%	52.3%	6.8%	52.0%	19.2%
60 - 69	n	323,139	187,210	24,880	46,118	5,646	210,752	25,495	77,627	25,207	208,949	16,616	200,591	39,762
	%		57.9%	13.3%	14.3%	12.2%	65.2%	12.1%	24.0%	32.5%	64.7%	8.0%	62.1%	19.8%
70 - 79	n	212,186	133,592	11,776	33,557	3,637	150,577	23,758	54,137	16,123	149,271	13,196	145,700	31,741
	%		63.0%	8.8%	15.8%	10.8%	71.0%	15.8%	25.5%	29.8%	70.3%	8.8%	68.7%	21.8%
80+	n	182,665	106,411	6,586	25,860	2,436	120,156	28,719	41,325	11,671	118,880	9,989	123,721	28,849
	%		58.3%	6.2%	14.2%	9.4%	65.8%	23.9%	22.6%	28.2%	65.1%	8.4%	67.7%	23.3%

ALP alkaline phosphatase; **ALT** alanine aminotransferase; **AST** aspartate aminotransferase; **GGT** gamma glutamyl transferase

For peer review only - http://bmjopen.bmj.com/site/about/guidelines.xhtml

Table 2. Annual frequency of testing per patient in those with at least 1 test

		ALT	AST	GGT	ALP	Bilirubin	Platelet count
Median (IQR) maximum number of tests		1 (1-2) 108	1 (1-1) 47	1 (1-1) 45	1 (1-2) 131	1 (1-2) 108	1 (1-2) 92
1	n	1,914,577	436,400	691,189	2,129,817	2,200,429	1,972,278
	%	74.2%	75.5%	75.6%	70.2%	74.1%	60.1%
2	n	457,080	99,123	155,168	610,628	528,607	849,020
	%	17.7%	17.1%	17.0%	20.1%	17.8%	25.9%
3	n	121,616	24,862	40,197	164,364	139,762	185,136
	%	4.7%	4.3%	4.4%	5.4%	4.7%	5.6%
4	n	39,173	8,279	13,221	61,498	45,583	153,538
	%	1.5%	1.4%	1.4%	2.0%	1.5%	4.7%
5	n	15,569	3,336	4,960	23,572	18,166	29,952
	%	0.6%	0.6%	0.5%	0.8%	0.6%	0.9%
6-10	n	22,702	4,909	7,084	32,002	26,262	73,577
	%	0.9%	0.8%	0.8%	1.1%	0.9%	2.2%
11+	n	9,349	1,400	2,610	11,234	10,248	18,286
	%	0.4%	0.2%	0.3%	0.4%	0.3%	0.6%

ALP alkaline phosphatase; **ALT** alanine aminotransferase; **AST** aspartate aminotransferase; **GGT** gamma glutamyl transferase

For peer review only

Risk factor subgroup analyses

The prevalence of liver marker testing over time by the subgroups (no liver risk factors, excess alcohol consumption and/or obesity) showed similar trends to those for the whole population and are shown in Supplementary figure S1 and Supplementary table S3.

People with type 2 diabetes had a notably higher prevalance of testing for all markers (e.g. in 2016 ALT measured in 68.8% of those with type 2 diabetes vs 15.3% and 21.9% of those with alcohol excess and obesity respectively. However, the rates of decline in measurement of AST and GGT were also faster in those with diabetes than the other groups; for AST falling from 24.3% in 2004 to 6.5% in 2016 vs 6.5% and 9.8% to 3.0% and 3.4% of those with alcohol excess and obesity respectively; and for GGT falling from 28.6% in 2004 to 13.1% in 2016 vs 9.7% and 11.8% to 7.3% and 7.7% of those with alcohol excess and obesity respectively.

People with no risk factors for liver disease had the lowest prevalence of liver marker testing for all markers, however did still follow the same trends over time – increasing for ALT, ALP, bilirubin and platelets, and falling for AST and GGT.

For peer review only

Discussion

We found that whilst the majority of liver blood markers have shown increased rates of use in general practice over the past 10 years there was wide variation by both marker and subgroups of the population. Most notably, the use of AST has fallen to only 2% per annum amongst all general practice users.

The striking finding that AST is now only measured in a fraction of the population has significant implications for policy and practice. Major international guidelines, including American, European and British[7,17,18] all utilize non-invasive markers for investigating liver disease at a community level. AST is a critical component of Fib-4 which has been suggested as a first line test; to rule out significant disease. The absence of AST as a routinely collected marker presents a major barrier to the current implementation of pathways that attend to the aforementioned guidelines. Furthermore, we found that <9% of abnormal ALT measurements also had an AST measured within a 6 week window.

The decision to prioritise ALT measurement over AST may have been driven by a push for efficiency savings[19] with ALT being considered more valuable as it is more liver specific. However AST may be a more sensitive indicator of chronic liver injury[20–22] especially when used as a ratio with ALT. In some regions an AST is automatically added if the ALT measure is abnormal to facilitate the AST/ALT ratio[23]. Over the 12year period examined nearly 40% of the population had at least one ALT measurement. This far exceeds the proportion of the known population dying prematurely of liver disease (estimated at 26,265 premature deaths in England in 2015-2017[24]), or the prevalence of recognised hepatic cirrhosis (estimated at 76.3 per 100,000 in 2001)[25]. Though the level of CLD in the UK is not known it is unlikely therefore that these tests are all done in those who have it or even are at high risk, and we therefore have to question why they are being performed and the opportunity cost it represents. Existing evidence suggests they are more often measured as part of routine monitoring than for CLD identification[26,27], and that discontinuation of such drugs rarely results[28]. If all these abnormalities were to be followed up (in accordance with existing guidance) there would be significant implications for downstream services. This includes the cost of a full liver screen, liver ultrasound and onward consultation and investigation in secondary care (e.g. national tariff for ultrasound scan £75.50, new patient consultant led hepatology outpatient appointment is £208.56[29]). Furthermore, there is growing evidence that in advanced liver disease many individuals have a normal ALT[10,30], so the growth in use of this marker as a trigger for further assessment may still not identify liver disease.

For peer review only

A more nuanced approach where non-invasive markers are targeted towards individuals with risk factors for CLD may be one solution. From a diagnostic perspective it increases the pre-test probability of having disease and indeed this approach has been shown to be cost effective regardless of choice of biomarker[31,32] and region studied[33]. Within CPRD those patients with risk factors for CLD, as expected, had higher proportions receiving liver markers assessment than those without risk factors. However, this was still very varied by 2016, with 70% of individuals with T2DM having an ALT measure that year, more than double those with obesity and nearly three times those with alcohol excess – with all three groups having similar proportions of abnormal results. Whilst AST testing was more frequent amongst those with risk factors than in those without it was still very low (<8% in all groups). Therefore, from an implementation perspective it would make sense to focus efforts of obtaining AST and ALT in these groups, appreciating as step change in management is needed.

The strengths of this population approach are driven by the use of a dataset known to be broadly representative of the UK population in terms of age, gender and geographical location with robust quality controls[14] and also the use of validated code lists for subgroup identification[34]. It is therefore reasonable to assume that our findings regarding the level of testing overall and in subgroups are representative of what is happening in the UK. A key limitation is the lack of information on the indication for testing or the resultant actions which clearly limits interpretation to some degree. Additionally, since this study only includes people who attend the GP, and some of the individuals at highest risk of CLD will not be attending, estimates of the proportion of tests which would be abnormal with more systematic testing may be less accurate.

In conclusion, large numbers of liver blood markers are being measured annually in UK primary care. At present, they are not suitable for risk stratifying high risk populations for CLD as the key element (AST) required to calculate non-invasive fibrosis markers is missing. However, the highest risk groups are receiving regular blood testing (69%% of those with diabetes and 22% of those with obesity) so routine or opportunistic risk stratification could be feasible with limited additional expense to the NHS.

Funding

This study was funded as part of a Medical Research Council Clinician Scientist award held by JRM (grant number MR/P008348/1).

For peer review only

Conflict of interest

Nil to declare.

Authorship

The authors confirm contribution to the paper as follows: study conception and design: JRM, JW, TCC; data collection: JRM; analysis and interpretation of results: JRM, PS, LB, JW, TCC, CC; draft manuscript preparation: JRM, PS, ING. All authors reviewed the results and approved the final version of the manuscript.

Data sharing

This data was extracting following approval from the Clinical Practice Research Datalink (CPRD, https://www.cprd.com/). Applications for access should be directed directly to CPRD.

For peer review only

References

1. Public Health England. *Liver Disease: Applying All Our Health [Internet]*. https://www.gov.uk/government/publications/liver-disease-applying-all-our-health/liver-disease-applying-all-ourhealth accessed 10.03.2021 https://www.gov.uk/government/publications/liver-disease-applying-all-our-health/liver-disease-applying-all-ourhealth accessed 10.03.2021

2. Public Health England. Liver Disease Profiles [Internet]. Accessed February 23, 2016. http://fingertips.phe.org.uk/profile/liver-disease accessed 25 June 2020

3. Public Health England. *The 2nd Atlas of Variation in Risk Factors and Healthcare for Liver Disease in England. Reducing Unwarranted Variation to Improve Health Outcomes and Value [Internet]*. http://tools.england.nhs.uk/images/LiverAtlas17/atlas.html; 2017. https://www.gov.uk/government/publications/liver-disease-applying-all-our-health/liver-disease-applying-all-ourhealth accessed 10.03.2021

4. Williams R, Aspinall R, Bellis M, et al. Addressing liver disease in the UK: a blueprint for attaining excellence in health care and reducing premature mortality from lifestyle issues of excess consumption of alcohol, obesity, and viral hepatitis. *The Lancet*. 2014;384(9958):1953-1997. http://linkinghub.elsevier.com/retrieve/pii/S0140673614618389

5. Davies S. Chief Medical Officer annual report 2011: volume 1, chapter 2c, p 163. *Dep Health Novemb 2012 Available Httpswwwgovukgovernmentuploadssystemuploadsattachmentdatafile141773CMOAnnualReport2011Chapter2cpdf Accessed 01072015*. Published online 2012.

6. Liver Disease: Today's complacency, tomorrow's catastrophe. All-Party Parliamentary Hepatology Group (APPHG) Inquiry into Improving Outcomes in Liver Disease. *Available HttpwwwiasorgukuploadsAPPHG20report20March20201420FINALpdf Accessed 01072015*. Published online 2014.

7. Newsome PN, Cramb R, Davison SM, et al. Guidelines on the management of abnormal liver blood tests. *Gut*. 2018;67(1):6-19.

8. National Institute for Health and Care Excellence. NICE guidelines [NG49] Non-alcoholic fatty liver disease (NAFLD): assessment and management. Published online July 2016. Accessed September 8, 2016. https://www.nice.org.uk/guidance/ng49

9. National Institute for Health and Care Excellence. NICE guidelines [NG50] Cirrhosis in over 16s: assessment and management. Published online July 2016. Accessed September 8, 2016. https://www.nice.org.uk/guidance/ng50

10. Chalmers J, Wilkes E, Harris R, et al. Development and implementation of a commissioned pathway for the identification and stratification of liver disease in the community. *Frontline Gastroenterol*. Published online June 26, 2019:flgastro-2019-101177. doi:10.1136/flgastro-2019-101177

For peer review only

11. Wai C-T, Greenson JK, Fontana RJ, et al. A simple noninvasive index can predict both significant fibrosis and cirrhosis in patients with chronic hepatitis C. *Hepatology*. 2003;38(2):518-526. http://dx.doi.org/10.1053/jhep.2003.50346

12. Vallet-Pichard A, Mallet V, Nalpas B, et al. FIB-4: An inexpensive and accurate marker of fibrosis in HCV infection. comparison with liver biopsy and fibrotest. *Hepatology*. 2007;46(1):32-36. doi:10.1002/hep.21669

13. Hydes T, Moore M, Stuart B, et al. Can routine blood tests be modelled to detect advanced liver disease in the community: model derivation and validation using UK primary and secondary care data. *BMJ Open*. 2021;11(2):e044952. doi:10.1136/bmjopen-2020-044952

14. Herrett E, Gallagher AM, Bhaskaran K, et al. Data Resource Profile: Clinical Practice Research Datalink (CPRD). *Int J Epidemiol*. 2015;44(3):827-836.

15. Herbert A, Wijlaars L, Zylbersztejn A, Cromwell D, Hardelid P. Data Resource Profile: Hospital Episode Statistics Admitted Patient Care (HES APC). *Int J Epidemiol*. 2017;46(4):1093-1093i. doi:10.1093/ije/dyx015

16. Padmanabhan S, Carty L, Cameron E, Ghosh RE, Williams R, Strongman H. Approach to record linkage of primary care data from Clinical Practice Research Datalink to other health-related patient data: overview and implications. *Eur J Epidemiol*. 2019;34(1):91-99. doi:10.1007/s10654-018-0442-4

17. Berzigotti A, Tsochatzis E, Boursier J, et al. EASL Clinical Practice Guidelines on non-invasive tests for evaluation of liver disease severity and prognosis – 2021 update. *J Hepatol*. Published online June 21, 2021. doi:10.1016/j.jhep.2021.05.025

18. Chalasani N, Younossi Z, Lavine JE, et al. The diagnosis and management of nonalcoholic fatty liver disease: Practice guidance from the American Association for the Study of Liver Diseases. *Hepatology*. 2018;67(1):328-357. doi:10.1002/hep.29367

19. Xu Q, Higgins T, Cembrowski GS. Limiting the Testing of AST: A Diagnostically Nonspecific Enzyme. *Am J Clin Pathol*. 2015;144(3):423-426. doi:10.1309/AJCPO47VAWYRIDHG

20. Daniel S, Ben-Menachem T, Vasudevan G, Ma CK, Blumenkehl M. Prospective evaluation of unexplained chronic liver transaminase abnormalities in asymptomatic and symptomatic patients. *Am J Gastroenterol*. 1999;94(10):3010-3014. doi:10.1016/S0002-9270(99)00507-9

21. Whitehead MW, Hawkes ND, Hainsworth I, Kingham JGC. A prospective study of the causes of notably raised aspartate aminotransferase of liver origin. *Gut*. 1999;45(1):129-133. doi:10.1136/gut.45.1.129

22. Lilford RJ, Bentham L, Girling A, et al. *Birmingham and Lambeth Liver Evaluation Testing Strategies (BALLETS): A Prospective Cohort Study*. NIHR Journals Library; 2013.

23. Dillon JF, Miller MH, Robinson EM, et al. Intelligent liver function testing (iLFT): A trial of automated diagnosis and staging of liver disease in primary care. *J Hepatol*. 2019;71(4):699-706.

For peer review only

24. Public Health Profiles. Accessed June 25, 2020. https://fingertips.phe.org.uk/profile/liver-disease

25. Fleming KM, Aithal GP, Solaymani-Dodaran M, Card TR, West J. Incidence and prevalence of cirrhosis in the United Kingdom, 1992–2001: a general population-based study. *J Hepatol*. 2008;49(5):732-738.

26. Standing HC, Jarvis H, Orr J, et al. GPs' experiences and perceptions of early detection of liver disease: a qualitative study in primary care. *Br J Gen Pr*. 2018;68(676):e743-e749.

27. Patel PJ, Banh X, Horsfall LU, et al. Underappreciation of non-alcoholic fatty liver disease by primary care clinicians: limited awareness of surrogate markers of fibrosis. *Intern Med J*. 2018;48(2):144-151.

28. Nakafero G, Grainge MJ, Card T, et al. What is the incidence of methotrexate or leflunomide discontinuation related to cytopenia, liver enzyme elevation or kidney function decline? *Rheumatology*. 2021;(keab254). doi:10.1093/rheumatology/keab254

29. UK Department of Health. National Cost Collection: National Schedule of NHS costs - Year 2019-20 - NHS trust and NHS foundation trusts. Availble at https://www.england.nhs.uk/national-cost-collection/.

30. Fracanzani AL, Valenti L, Bugianesi E, et al. Risk of severe liver disease in nonalcoholic fatty liver disease with normal aminotransferase levels: A role for insulin resistance and diabetes. *Hepatology*. 2008;48(3):792-798. doi:https://doi.org/10.1002/hep.22429

31. Srivastava A, Jong S, Gola A, et al. Cost-comparison analysis of FIB-4, ELF and fibroscan in community pathways for non-alcoholic fatty liver disease. *BMC Gastroenterol*. 2019;19(1):122. doi:10.1186/s12876-019-1039-4

32. Tanajewski L, Harris R, Harman DJ, et al. Economic evaluation of a community-based diagnostic pathway to stratify adults for non-alcoholic fatty liver disease: a Markov model informed by a feasibility study. *BMJ Open*. 2017;7(6):e015659. doi:10.1136/bmjopen-2016-015659

33. Serra-Burriel M, Graupera I, Torán P, et al. Transient elastography for screening of liver fibrosis: cost-effectiveness analysis from six prospective cohorts in Europe and Asia. *J Hepatol*. 2019;71(6):1141-1151.

34. Ratib S, West J, Crooks CJ, Fleming KM. Diagnosis of Liver Cirrhosis in England, a Cohort Study, 1998-2009: A Comparison With Cancer. *Am J Gastroenterol*. 2014;109(2):190-198. http://dx.doi.org/10.1038/ajg.2013.405

For peer review only

Figure 1 Prevalence of liver enzyme testing amongst adults over time

ALP alkaline phosphatase; **ALT** alanine aminotransferase; **AST** aspartate aminotransferase; **GGT** gamma glutamyl transferase

Figure 2 Prevalence of abnormal values of liver blood tests in adults over time

ALP alkaline phosphatase; **ALT** alanine aminotransferase; **AST** aspartate aminotransferase; **GGT** gamma glutamyl transferase

For peer review only

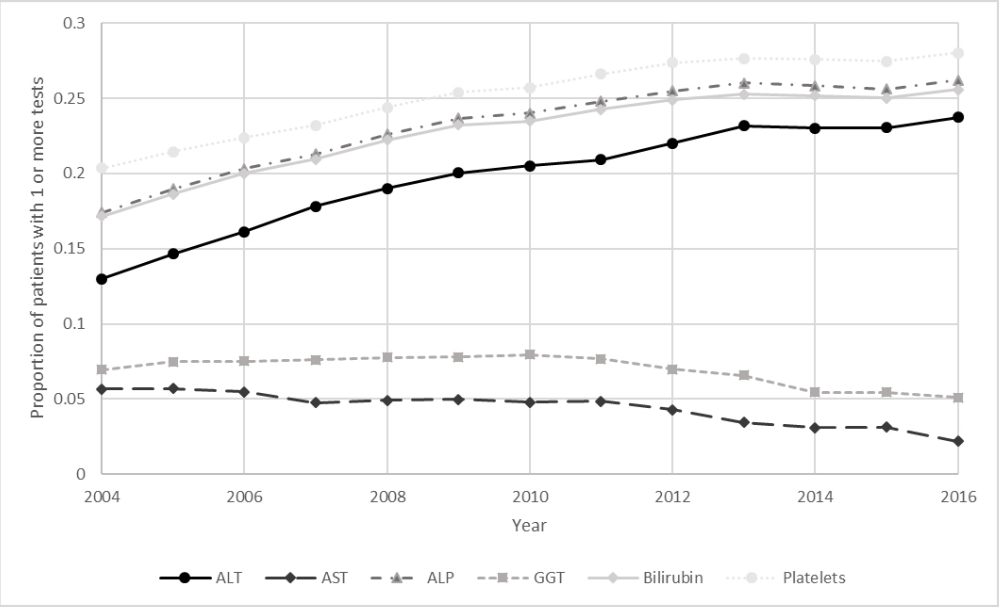

Figure 1 Prevalence of liver enzyme testing amongst adults over time

ALP alkaline phosphatase; ALT alanine aminotransferase; AST aspartate aminotransferase; GGT gamma glutamyl transferase

456x277mm (59 x 59 DPI)

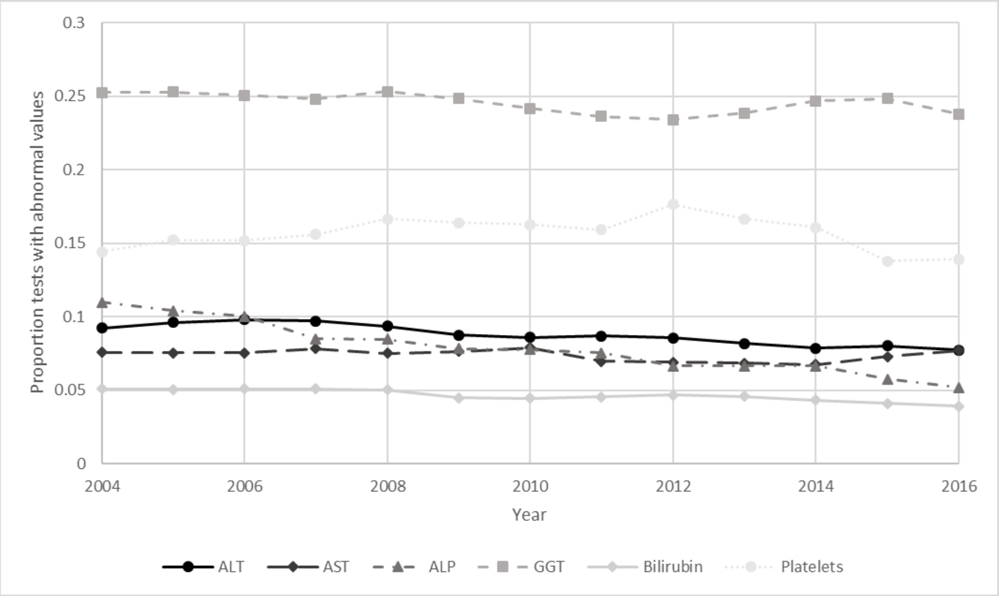

Figure 2 Prevalence of abnormal values of liver blood tests in adults over time

ALP alkaline phosphatase; ALT alanine aminotransferase; AST aspartate aminotransferase; GGT gamma glutamyl transferase

459x274mm (59 x 59 DPI)

Supplementary Table S1 – Codes

Liver blood tests and their values were identified from the CPRD "test" dataset using the following entity file codes: ALT = 155; AST = 156; ALP = 153; GGT = 172; Bilirubin = 158; Platelet count = 189.

Type 2 diabetes

C100112	Non-insulin dependent diabetes mellitus	C109200	Non-insulin-dependent diabetes mellitus with neuro comps
C107400	NIDDM with peripheral circulatory disorder	C109211	Type II diabetes mellitus with neurological complications
C109.00	Non-insulin dependent diabetes mellitus	C109212	Type 2 diabetes mellitus with neurological complications
C109.11	NIDDM - Non-insulin dependent diabetes mellitus	C109300	Non-insulin-dependent diabetes mellitus with multiple comps
C109.12	Type 2 diabetes mellitus	C109312	Type 2 diabetes mellitus with multiple complications
C109.13	Type II diabetes mellitus	C109400	Non-insulin dependent diabetes mellitus with ulcer
C109000	Non-insulin-dependent diabetes mellitus with renal comps	C109411	Type II diabetes mellitus with ulcer
C109011	Type II diabetes mellitus with renal complications	C109412	Type 2 diabetes mellitus with ulcer
C109012	Type 2 diabetes mellitus with renal complications	C109500	Non-insulin dependent diabetes mellitus with gangrene
C109100	Non-insulin-dependent diabetes mellitus with ophthalm comps	C109511	Type II diabetes mellitus with gangrene
C109111	Type II diabetes mellitus with ophthalmic complications	C109512	Type 2 diabetes mellitus with gangrene
C109112	Type 2 diabetes mellitus with ophthalmic complications	C109600	Non-insulin-dependent diabetes mellitus with retinopathy

Obesity = BMI>30. Values obtained from entity file: weight =13 and height = 14.
Calculate BMI for every available weight measurement (using single height measurement). If BMI > 30 at any stage up to end of follow-up for individual patient then include in Obesity population. Index date is first occurrence of BMI >30.

Cirrhosis: Identified from READ codes/terms

7609	Open operations on oesophageal varices	J612.12	Laennec's cirrhosis
7609300	Local ligation of oesophageal varices	J615.00	Cirrhosis - non alcoholic
7609400	Open injection sclerotherapy to oesophageal varices	J615.11	Portal cirrhosis
7609y11	Tanner devascularisation for bleeding varices	J615100	Multilobular portal cirrhosis
7609z00	Open operation on oesophageal varices NOS	J615300	Diffuse nodular cirrhosis
760C300	Fibreoptic endoscopic injection sclerotherapy oesoph varices	J615400	Fatty portal cirrhosis
760C500	Fibreoptic endoscopic banding of oesophageal varices	J615500	Hypertrophic portal cirrhosis
760F300	Rigid oesophagoscopic injection sclerotherapy oesoph varices	J615600	Capsular portal cirrhosis
760F400	Rigid oesophagoscopic banding of oesophageal varices	J615700	Cardiac portal cirrhosis
761D800	Fibreopt endoscop rubber band ligation of upper GIT varices	J615800	Juvenile portal cirrhosis
C310400	Glycogenosis with hepatic cirrhosis	J615812	Indian childhood cirrhosis

For peer review only

C350012	Pigmentary cirrhosis of liver	J615C00	Xanthomatous portal cirrhosis
G85..11	Oesophageal varices	J615D00	Bacterial portal cirrhosis
G850.00	Oesophageal varices with bleeding	J615H00	Infectious cirrhosis NOS
G851.00	Oesophageal varices without bleeding	J615y00	Portal cirrhosis unspecified
G852.00	Oesophageal varices in diseases EC	J615z00	Non-alcoholic cirrhosis NOS
G852000	Oesophageal varices with bleeding in diseases EC	J615z11	Macronodular cirrhosis of liver
G852100	Oesophageal varices without bleeding in diseases EC	J615z12	Cryptogenic cirrhosis of liver
G852200	Oesophageal varices in cirrhosis of the liver	J615z13	Cirrhosis of liver NOS
G852300	Oesophageal varices in alcoholic cirrhosis of the liver	J616.00	Biliary cirrhosis
G852z00	Oesophageal varices in diseases EC NOS	J616100	Secondary biliary cirrhosis
G857.00	Gastric varices	J616200	Biliary cirrhosis of children
G858.00	Oesophageal varices NOS	J616z00	Biliary cirrhosis NOS
J61..00	Cirrhosis and chronic liver disease	J623.00	Portal hypertension
J612.00	Alcoholic cirrhosis of liver	J635600	Toxic liver disease with fibrosis and cirrhosis of liver
J612.11	Florid cirrhosis	Jyu7100	[X]Other and unspecified cirrhosis of liver
**Alcohol excess:** Identified from units per week from entity file (entity=5) and READ codes/terms			
136K.00	Alcohol intake above recommended sensible limits	E231.00	Chronic alcoholism
136S.00	Hazardous alcohol use	E231z00	Chronic alcoholism NOS
136T.00	Harmful alcohol use	E23z.00	Alcohol dependence syndrome NOS
136W.00	Alcohol misuse	E250.00	Nondependent alcohol abuse
8H7p.00	Referral to community alcohol team	E250000	Nondependent alcohol abuse, unspecified
9NN2.00	Under care of community alcohol team	E250200	Nondependent alcohol abuse, episodic
9k1..00	Alcohol misuse - enhanced services administration	E250z00	Nondependent alcohol abuse NOS
9k1A.00	Brief intervention for excessive alcohol consumption completed	Eu10211	[X]Alcohol addiction
E23..00	Alcohol dependence syndrome	Eu10212	[X]Chronic alcoholism
E23..11	Alcoholism	ZV11300	[V]Personal history of alcoholism
E23..12	Alcohol problem drinking		

For peer review only

Supplementary Table S2 – Total numbers of tests measured and individuals being tested

	2004	2005	2006	2007	2008	2009	2010	2011	2012	2013	2014	2015	2016
**ALT**													
Total tests	145,762	182,250	220,863	264,573	299,956	336,059	349,857	355,185	377,633	379,820	336,022	274,827	199,196
Total people tested	101,485	126,562	152,516	183,196	206,761	228,380	239,920	246,920	261,544	264,206	233,037	193,654	141,885
**AST**													
Total tests	62,414	69689	72,998	69218	75,502	81218	78,629	79997	71,305	53,714	44,064	36,542	18,363
Total people tested	44,294	49158	51,781	48791	53,406	56756	56,085	57243	50,856	39,178	31,411	26,275	13,075
**ALP**													
Total tests	199,350	241693	288,224	326253	368,076	411994	426,350	442311	461,329	454,319	405,753	332,655	236,886
Total people tested	136,121	163894	192,150	218956	245,608	269713	280,957	292771	302,822	296,931	261,378	215,265	156,549
**GGT**													
Total tests	78,572	92452	102,634	111127	119,522	127386	132,905	127210	116,707	102,085	74,190	61,516	40,953
Total people tested	54,346	64692	71,192	78118	84,354	88723	93,147	90618	82,859	75,125	55,022	45,726	30,507
**Bilirubin**													
Total tests	192,632	231683	275,631	311066	350,373	388612	399,189	411125	426,237	412,887	366,861	299,687	215,528
Total people tested	134,046	160842	189,233	215296	241,713	264757	274,782	286590	295,722	288,212	254,867	210,081	152,916
**Platelet count**													
Total tests	269,019	318699	368,616	419187	475,732	524655	540,431	555732	589,239	557,555	495,158	389,745	279,714
Total people tested	158,987	185336	211,828	238400	265,257	289243	300,868	314311	324,861	315,432	279,168	230,590	167,506

For peer review only

Supplementary Table S3 Prevalence of liver blood markers over time

	2004	2005	2006	2007	2008	2009	2010	2011	2012	2013	2014	2015	2016
**ALT**													
All	13.0%	14.7%	16.1%	17.8%	19.0%	20.0%	20.5%	20.9%	22.0%	23.2%	23.0%	23.1%	23.7%
	101,485	126,562	152,516	183,196	206,761	228,380	239,920	246,920	261,544	264,206	233,037	193,654	141,885
No risk	10.2%	11.5%	12.3%	13.7%	14.6%	15.4%	15.6%	15.9%	16.7%	17.5%	17.4%	17.3%	17.9%
factor	58,099	71,085	82,630	97,925	109,206	119,415	122,910	125,077	131,327	131,491	115,211	93,323	69,323
Alcohol	15.3%	17.2%	19.2%	21.5%	23.1%	24.4%	25.1%	25.6%	27.1%	28.3%	28.0%	27.4%	28.3%
excess	15,571	19,838	24,616	30,304	34,840	38,992	41,558	42,907	45,955	46,207	41,346	34,669	23,270
Obesity	21.9%	24.6%	27.2%	29.2%	30.6%	31.6%	32.4%	32.5%	33.9%	35.4%	35.0%	35.1%	35.7%
	25,460	33,357	42,787	53,140	61,862	70,159	76,530	80,517	86,275	88,493	79,136	68,224	50,029
Type 2	56.1%	61.0%	64.4%	67.9%	68.4%	68.1%	68.9%	67.4%	68.5%	70.7%	68.4%	68.6%	68.8%
diabetes	10,271	13,741	17,680	21,964	24,670	27,140	29,564	31,100	33,335	34,909	31,408	27,066	20,317
**AST**													
All	5.7%	5.7%	5.5%	4.7%	4.9%	5.0%	4.8%	4.8%	4.3%	3.4%	3.1%	3.1%	2.2%
	44,294	49,158	51,781	48,791	53,406	56,756	56,085	57,243	50,856	39,178	31,411	26,275	13,075
No risk	4.4%	4.5%	4.1%	3.6%	3.7%	3.7%	3.6%	3.7%	3.3%	2.6%	2.3%	2.3%	1.6%
factor	25,237	27,674	27,556	25,603	27,614	28,920	28,504	29,130	25,902	19,882	15,406	12,526	6,017
Alcohol	6.5%	6.5%	6.5%	5.5%	5.6%	5.7%	5.5%	5.5%	4.8%	3.9%	3.6%	3.8%	3.0%
excess	6,672	7,446	8,311	7,708	8,497	9,129	9,123	9,214	8,206	6,298	5,368	4,791	2,503
Obesity	9.8%	9.7%	9.6%	8.2%	8.4%	8.3%	7.8%	7.6%	6.6%	5.3%	4.8%	4.8%	3.4%
	11,411	13,137	15,047	14,942	17,073	18,511	18,354	18,929	16,709	13,124	10,921	9,327	4,740
Type 2	24.3%	23.6%	22.8%	18.9%	18.9%	18.4%	16.8%	16.0%	13.6%	10.3%	9.1%	9.6%	6.5%
diabetes	4,444	5,309	6,257	6,104	6,808	7,318	7,228	7,387	6,599	5,099	4,155	3,780	1,919
**ALP**													
All	17.4%	19.0%	20.3%	21.3%	22.6%	23.7%	24.0%	24.8%	25.5%	26.0%	25.8%	25.6%	26.2%
	136,121	163,894	192,150	218,956	245,608	269,713	280,957	292,771	302,822	296,931	261,378	215,265	156,549
No risk	13.8%	14.9%	15.6%	16.4%	17.4%	18.2%	18.3%	19.0%	19.5%	19.9%	19.7%	19.4%	19.9%
factor	78,382	92,690	104,627	117,407	129,995	141,235	144,684	149,282	153,272	149,168	130,327	104,727	76,926

For peer review only

Alcohol excess	20.0%	21.7%	23.8%	25.0%	26.6%	27.9%	28.6%	29.4%	30.5%	31.0%	30.7%	29.9%	31.1%
	20,355	25,039	30,447	35,275	40,123	44,632	47,212	49,230	51,624	50,602	45,304	37,836	25,565
Obesity	29.5%	31.9%	34.3%	35.1%	36.8%	37.7%	38.1%	38.7%	39.2%	39.7%	39.1%	38.8%	39.2%
	34,314	43,222	53,982	63,964	74,331	83,756	89,904	95,755	99,898	99,094	88,395	75,373	54,922
Type 2 diabetes	73.5%	77.4%	79.6%	80.4%	81.0%	80.6%	80.2%	79.3%	78.6%	78.2%	75.5%	75.1%	75.0%
	13,458	17,429	21,845	26,018	29,210	32,115	34,420	36,568	38,245	38,590	34,647	29,619	22,161
**GGT**													
All	7.0%	7.5%	7.5%	7.6%	7.8%	7.8%	8.0%	7.7%	7.0%	6.6%	5.4%	5.4%	5.1%
	54,346	64,692	71,192	78,118	84,354	88,723	93,147	90,618	82,859	75,125	55,022	45,726	30,507
No risk factor	5.3%	5.6%	5.5%	5.5%	5.6%	5.6%	5.7%	5.5%	5.0%	4.7%	3.9%	3.9%	3.7%
	29,868	34,865	36,661	39,595	42,214	43,632	45,103	43,616	39,378	35,625	25,434	20,846	14,408
Alcohol excess	9.7%	10.3%	10.8%	11.0%	11.3%	11.5%	11.7%	11.2%	10.4%	9.6%	8.3%	7.9%	7.3%
	9,837	11,909	13,845	15,572	17,021	18,307	19,323	18,790	17,571	15,738	12,271	9,922	5,971
Obesity	11.8%	12.8%	12.9%	12.6%	12.8%	12.6%	12.8%	12.1%	10.9%	10.2%	8.4%	8.5%	7.7%
	13,754	17,296	20,350	23,024	25,877	27,954	30,300	29,929	27,680	25,385	19,030	16,469	10,731
Type 2 diabetes	28.6%	29.3%	28.0%	27.1%	26.2%	25.1%	25.3%	23.7%	20.7%	19.2%	14.8%	15.1%	13.1%
	5,232	6,601	7,693	8,757	9,456	10,007	10,855	10,946	10,061	9,484	6,796	5,942	3,879
**Bilirubin**													
All	17.2%	18.6%	20.0%	20.9%	22.2%	23.2%	23.5%	24.3%	24.9%	25.3%	25.2%	25.0%	25.6%
	134,046	160,842	189,233	215,296	241,713	264,757	274,782	286,590	295,722	288,212	254,867	210,081	152,916
No risk factor	13.5%	14.6%	15.3%	16.1%	17.0%	17.8%	17.8%	18.4%	18.9%	19.2%	19.1%	18.8%	19.3%
	76,925	90,589	102,695	114,978	127,452	137,983	140,694	145,318	148,912	143,914	126,200	101,520	74,600
Alcohol excess	19.8%	21.5%	23.5%	24.7%	26.4%	27.6%	28.2%	29.0%	30.0%	30.3%	30.2%	29.4%	30.7%
	20,174	24,766	30,144	34,890	39,753	44,116	46,549	48,609	50,790	49,536	44,537	37,217	25,195
Obesity	29.1%	31.4%	33.9%	34.7%	36.3%	37.2%	37.4%	38.0%	38.5%	38.7%	38.3%	38.0%	38.5%
	33,896	42,574	53,306	63,125	73,377	82,584	88,397	94,097	97,916	96,601	86,706	73,942	53,945
Type 2 diabetes	73.2%	76.9%	79.1%	79.9%	80.5%	80.0%	79.5%	78.7%	77.7%	77.0%	74.8%	74.5%	74.4%
	13,402	17,306	21,709	25,850	29,030	31,878	34,108	36,294	37,812	38,009	34,346	29,378	21,978
**Platelets**													

For peer review only

All	20.3% 158,987	21.5% 185,336	22.4% 211,828	23.2% 238,400	24.4% 265,257	25.4% 289,243	25.7% 300,868	26.6% 314,311	27.4% 324,861	27.6% 315,432	27.6% 279,168	27.5% 230,590	28.0% 167,506
No risk factor	18.3% 104,008	19.1% 118,472	19.5% 130,674	20.1% 144,262	21.0% 157,524	21.8% 168,847	21.8% 172,317	22.5% 177,447	23.0% 180,888	23.1% 173,265	22.9% 151,378	22.4% 121,000	22.9% 88,638
Alcohol excess	18.6% 18,994	19.9% 22,933	21.4% 27,424	22.5% 31,684	24.0% 36,207	25.2% 40,327	26.0% 42,991	27.0% 45,170	28.4% 48,143	29.0% 47,396	29.1% 42,957	29.0% 36,654	30.0% 24,657
Obesity	28.7% 33,393	30.6% 41,452	32.4% 50,954	33.1% 60,244	34.7% 70,172	35.9% 79,767	36.5% 86,087	37.5% 92,765	38.3% 97,383	38.6% 96,496	38.6% 87,227	38.9% 75,562	39.4% 55,216
Type 2 diabetes	47.9% 8,764	51.1% 11,511	54.4% 14,932	56.1% 18,153	58.3% 21,031	59.5% 23,689	61.9% 26,553	63.2% 29,146	64.0% 31,107	64.4% 31,793	63.7% 29,233	65.3% 25,752	66.3% 19,593

For peer review only

Supplementary Figure S1 Prevalence of liver marker testing over time by the subgroups: no liver risk factors, Type 2 diabetes, excess alcohol consumption and obesity

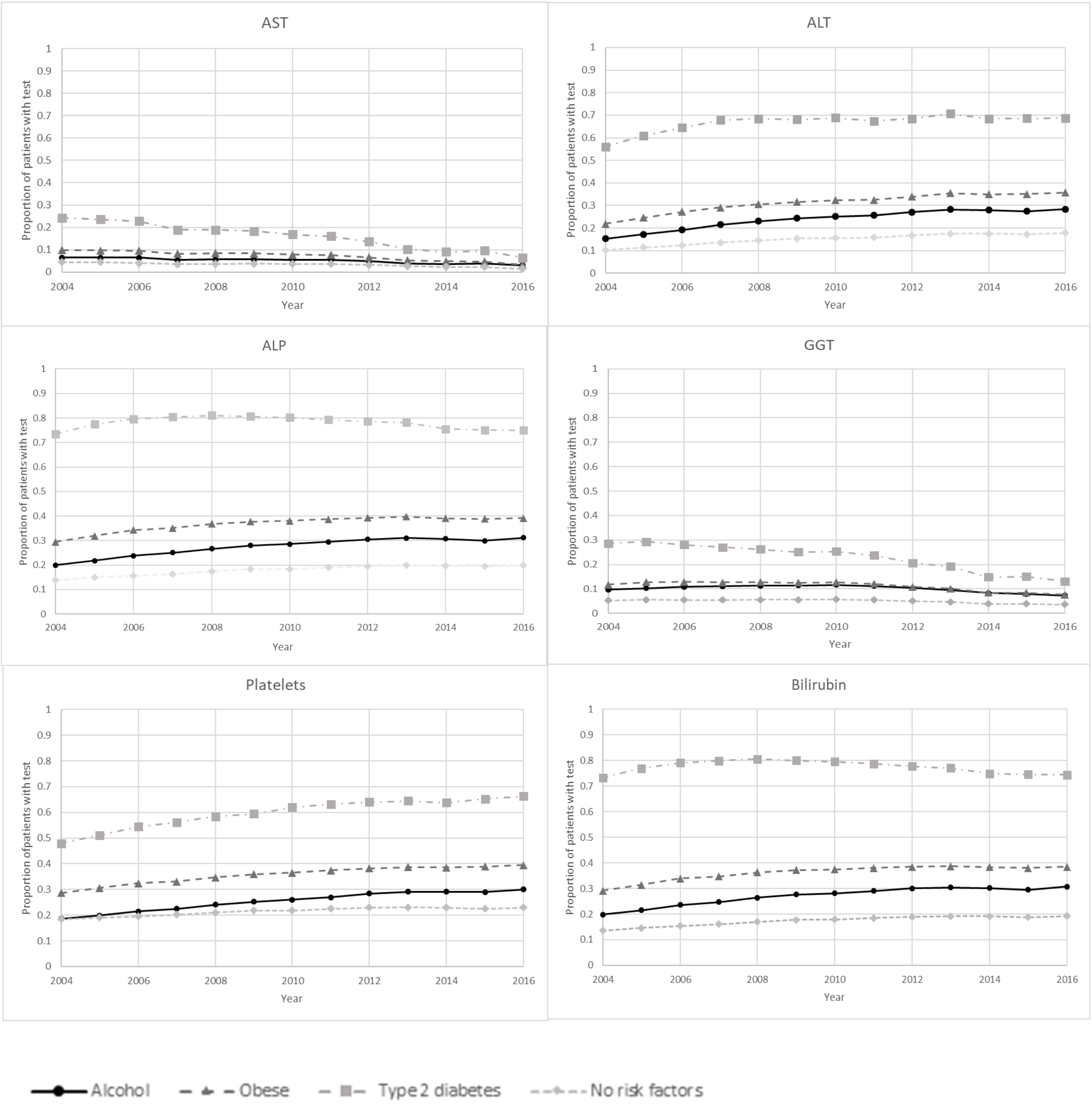

STROBE Statement—checklist of items that should be included in reports of observational studies

	Item No	Recommendation	
**Title and abstract**	1	(*a*) Indicate the study's design with a commonly used term in the title or the abstract	1
		(*b*) Provide in the abstract an informative and balanced summary of what was done and what was found	2
**Introduction**			
Background/rationale	2	Explain the scientific background and rationale for the investigation being reported	4
Objectives	3	State specific objectives, including any prespecified hypotheses	4
**Methods**			
Study design	4	Present key elements of study design early in the paper	5
Setting	5	Describe the setting, locations, and relevant dates, including periods of recruitment, exposure, follow-up, and data collection	5
Participants	6	(*a*) *Cohort study*—Give the eligibility criteria, and the sources and methods of selection of participants. Describe methods of follow-up<br>*Case-control study*—Give the eligibility criteria, and the sources and methods of case ascertainment and control selection. Give the rationale for the choice of cases and controls<br>*Cross-sectional study*—Give the eligibility criteria, and the sources and methods of selection of participants	5
		(*b*) *Cohort study*—For matched studies, give matching criteria and number of exposed and unexposed<br>*Case-control study*—For matched studies, give matching criteria and the number of controls per case	NA
Variables	7	Clearly define all outcomes, exposures, predictors, potential confounders, and effect modifiers. Give diagnostic criteria, if applicable	5/6
Data sources/ measurement	8*	For each variable of interest, give sources of data and details of methods of assessment (measurement). Describe comparability of assessment methods if there is more than one group	5/6
Bias	9	Describe any efforts to address potential sources of bias	5/6
Study size	10	Explain how the study size was arrived at	5
Quantitative variables	11	Explain how quantitative variables were handled in the analyses. If applicable, describe which groupings were chosen and why	6
Statistical methods	12	(*a*) Describe all statistical methods, including those used to control for confounding	6
		(*b*) Describe any methods used to examine subgroups and interactions	6
		(*c*) Explain how missing data were addressed	
		(*d*) *Cohort study*—If applicable, explain how loss to follow-up was addressed<br>*Case-control study*—If applicable, explain how matching of cases and controls was addressed<br>*Cross-sectional study*—If applicable, describe analytical methods taking account of sampling strategy	NA
		(*e*) Describe any sensitivity analyses	6

Continued on next page

For peer review only - http://bmjopen.bmj.com/site/about/guidelines.xhtml

**Results**			
Participants	13*	(a) Report numbers of individuals at each stage of study—eg numbers potentially eligible, examined for eligibility, confirmed eligible, included in the study, completing follow-up, and analysed	7
		(b) Give reasons for non-participation at each stage	7
		(c) Consider use of a flow diagram	NA
Descriptive data	14*	(a) Give characteristics of study participants (eg demographic, clinical, social) and information on exposures and potential confounders	7
		(b) Indicate number of participants with missing data for each variable of interest	7
		(c) *Cohort study*—Summarise follow-up time (eg, average and total amount)	7
Outcome data	15*	*Cohort study*—Report numbers of outcome events or summary measures over time	7/8
		Case-control study—Report numbers in each exposure category, or summary measures of exposure	
		Cross-sectional study—Report numbers of outcome events or summary measures	
Main results	16	(*a*) Give unadjusted estimates and, if applicable, confounder-adjusted estimates and their precision (eg, 95% confidence interval). Make clear which confounders were adjusted for and why they were included	7/8
		(*b*) Report category boundaries when continuous variables were categorized	7/8
		(*c*) If relevant, consider translating estimates of relative risk into absolute risk for a meaningful time period	
Other analyses	17	Report other analyses done—eg analyses of subgroups and interactions, and sensitivity analyses	8
**Discussion**			
Key results	18	Summarise key results with reference to study objectives	9
Limitations	19	Discuss limitations of the study, taking into account sources of potential bias or imprecision. Discuss both direction and magnitude of any potential bias	10
Interpretation	20	Give a cautious overall interpretation of results considering objectives, limitations, multiplicity of analyses, results from similar studies, and other relevant evidence	9/10
Generalisability	21	Discuss the generalisability (external validity) of the study results	10
**Other information**			
Funding	22	Give the source of funding and the role of the funders for the present study and, if applicable, for the original study on which the present article is based	10

*Give information separately for cases and controls in case-control studies and, if applicable, for exposed and unexposed groups in cohort and cross-sectional studies.

Note: An Explanation and Elaboration article discusses each checklist item and gives methodological background and published examples of transparent reporting. The STROBE checklist is best used in conjunction with this article (freely available on the Web sites of PLoS Medicine at http://www.plosmedicine.org/, Annals of Internal Medicine at http://www.annals.org/, and Epidemiology at http://www.epidem.com/). Information on the STROBE Initiative is available at www.strobe-statement.org.

For peer review only

BMJ Open

Liver blood marker testing in UK primary care: a UK wide cohort study, 2004-2016

Journal:	*BMJ Open*
Manuscript ID	bmjopen-2021-058967.R1
Article Type:	Original research
Date Submitted by the Author:	01-Mar-2022
Complete List of Authors:	Scutt, Polly; University of Nottingham, Stroke, Division of Clinical Neuroscience<br>Ban, Lu; University of Nottingham<br>Card, Tim; University of Nottingham<br>Crooks, Colin; University of Nottingham, Epidmiology and Public Health<br>Guha, Neil; University of Nottingham<br>West, Joe; University of Nottingham<br>Morling, Joanne; University of Nottingham
**Primary Subject Heading**:	Gastroenterology and hepatology
Secondary Subject Heading:	General practice / Family practice
Keywords:	Adult gastroenterology < GASTROENTEROLOGY, Health policy < HEALTH SERVICES ADMINISTRATION & MANAGEMENT, PREVENTIVE MEDICINE, PRIMARY CARE

SCHOLARONE™ Manuscripts

I, the Submitting Author has the right to grant and does grant on behalf of all authors of the Work (as defined in the below author licence), an exclusive licence and/or a non-exclusive licence for contributions from authors who are: i) UK Crown employees; ii) where BMJ has agreed a CC-BY licence shall apply, and/or iii) in accordance with the terms applicable for US Federal Government officers or employees acting as part of their official duties; on a worldwide, perpetual, irrevocable, royalty-free basis to BMJ Publishing Group Ltd ("BMJ") its licensees and where the relevant Journal is co-owned by BMJ to the co-owners of the Journal, to publish the Work in this journal and any other BMJ products and to exploit all rights, as set out in our licence.

The Submitting Author accepts and understands that any supply made under these terms is made by BMJ to the Submitting Author unless you are acting as an employee on behalf of your employer or a postgraduate student of an affiliated institution which is paying any applicable article publishing charge ("APC") for Open Access articles. Where the Submitting Author wishes to make the Work available on an Open Access basis (and intends to pay the relevant APC), the terms of reuse of such Open Access shall be governed by a Creative Commons licence – details of these licences and which Creative Commons licence will apply to this Work are set out in our licence referred to above.

Other than as permitted in any relevant BMJ Author's Self Archiving Policies, I confirm this Work has not been accepted for publication elsewhere, is not being considered for publication elsewhere and does not duplicate material already published. I confirm all authors consent to publication of this Work and authorise the granting of this licence.

For peer review only

Liver blood marker testing in UK primary care: a UK wide cohort study, 2004-2016

For peer review only

[Liver blood tests in UK primary care]

Scutt Polly[1], Ban Lu[1,2], Card Tim C[1,3], Crooks Colin[3,4], Guha I Neil[3,4], West Joe[1,3], Morling Joanne R[1,3]

1. Population and Lifespan Sciences, School of Medicine, University of Nottingham, Clinical Sciences Building 2, City Hospital, Nottingham, UK, NG5 1PB
2. Evidera (European Office),R1 The Ark, 201 Talgarth Rd, Hammersmith, London, UK, W6 8BJ
3. NIHR Nottingham Biomedical Research Centre (BRC), Nottingham University Hospitals NHS Trust and the University of Nottingham, Nottingham, UK, NG7 2UH
4. Nottingham Digestive Diseases Centre (NDDC), School of Medicine, University of Nottingham, Nottingham, UK, NG7 2UH

Corresponding author: Dr Jo Morling

Population and Lifespan Sciences, School of Medicine, University of Nottingham,

Clinical Sciences Building 2, City Hospital, Nottingham, UK, NG5 1PB Joanne.morling@nottingham.ac.uk

Words: 2,046

Keywords: screening, early diagnosis, aspartate aminotransferase, policy, population

Summary/Abstract

Objective

We aimed to determine i) the temporal trends of liver enzyme testing in UK general practice and ii) how these vary amongst different subgroups at risk of chronic liver disease (CLD).

Design

Retrospective cohort study

Setting

UK primary care database (Clinical Practice Research Datalink (CPRD)), 2004-2016

Participants:

Patients aged 18 years or over, registered in the CPRD from 1st January 2004 to 31st December 2016,

Outcome measures

The frequency of testing recorded within the study period in general practice was calculated for: Alanine aminotransferase (ALT); Aspartate aminotransferase (AST); Gamma glutamyl transferase (GTT); Alkaline phosphatase (ALP); Bilirubin and platelets. Analyses were conducted in subgroups of patients at high risk of developing liver disease.

Results

The study cohort included 2,912,066 individuals with median follow-up of 3.2 years. The proportion of patients with at least one measurement for ALT, ALP, Bilirubin or platelet test gradually increased over the course of the study period and fell for AST and GGT. By 2016 the proportion of the population receiving one of more tests in that year was: platelet count 28.0%, ALP 26.2%, bilirubin 25.6%, ALT 23.7%, GGT 5.1% and AST 2.2%. Those patients with risk factors for CLD had higher proportions receiving liver marker assessments than those without risk factors.

For peer review only

Conclusions

The striking finding that AST is now only measured in a fraction of the population has significant implications for routine guidance which frequently expects it. A more nuanced approach where non-invasive markers are targeted towards individuals with risk factors for CLD may be a solution.

[word count = 252]

Article summary

Strengths and limitations of this study

- Sampling frame: a significant strength is the use of a large national dataset (>15mill people).
- Data quality: the dataset used (CPRD) known to be representative of the UK population in terms of age, gender and geographical location with robust quality controls.
- Data validity: previously validated code lists were used for the identification of subgroups.
- A key limitation is the lack of information on the indication for testing or the resultant actions which limits interpretation to some degree.
- Since this study only includes people who attend the GP, and some of the individuals at highest risk of CLD will not be attending. Therefore, we underestimate the proportions potentially identified is systematic testing was employed.

For peer review only

Introduction

In the UK liver disease is a significant and growing burden on the National Health Service (NHS) and is the United Kingdom's third most common cause of premature mortality[1]; between 2015 and 2017 it caused 26,265 premature deaths in England alone[2]. It is also a significant source of healthcare inequity, with the median age of death differing by 9 years between the most and least deprived quintiles[3]. There has been a 400% increase in liver disease mortality in the population as a whole since 1970 and nearly 500% increases in mortality observed in working age populations over in this period[4].

Three independent reports since 2014 have highlighted the need for the early detection of chronic liver disease (CLD) including the Chief Medical Officer report (2012)[5], the All-Party Parliamentary Hepatology Group Inquiry[6] and the Lancet commission[4], in order to allow intervention and change the course of the disease. A number of organizations have now developed guidance advocating the use of non-invasive fibrosis markers in risk stratification[7–9]. Despite this, many existing community diagnostic pathways for detection and onward referral of suspected CLD are based on traditional liver enzyme tests which lack accuracy and result in delays to diagnosis[10].

The optimal non-invasive fibrosis marker is yet to be determined, however there are simple algorithms involving easily accessible measures such as AST and platelets that can be conducted in primary care, e.g. aspartate to platelet ratio index (APRI)[11], Fibrosis-4 score (FIB4)[12], and CIRRUS[13]. However, there is little understanding about how liver blood tests are currently used in UK general practice in order to support the implementation of changing practice and policy.

Given the rising prevalence of lifestyle related CLD and growing knowledge of non-invasive fibrosis measures one could hypothesise that there should have been a shift away from traditional liver blood testing over time (shifting to non-invasive assessment). The aim of this study was to determine i) the temporal trends of liver blood testing in UK general practice and ii) how these vary amongst different subgroups at risk of CLD.

For peer review only

Materials and Methods

Data source

A population-based cohort study was conducted using the Clinical Practice Research Datalink (CPRD). The CPRD contains primary care data on 15.5 million people from 734 practices in the UK and is considered representative of the UK population[14]. Data are anonymised at patient and practice level and contain information on patient demographics, consultations, diagnoses, referrals and prescriptions. Clinical information is entered using READ codes which was a standard clinical terminology system used in the UK. For a subset of English practices (58% of UK CPRD practices), primary care data can be linked with the Hospital Episode Statistics (HES) dataset containing information for all hospital admissions[15,16]. The population for this study consists only of patients from these practices eligible for linkage with the HES dataset. This was a fully anonymised databased study not requiring ethical approval. This use of the data for this study was approved by the Independent Scientific Advisory Committee (ISAC) for CPRD and the Medicines and Healthcare products Regulatory Agency (MHRA) and assigned reference Protocol 19_256.

Study Population

Patients aged 18 years or over, registered in the CPRD from 1st January 2004 to 31st December 2016, and having at least one day of registration with a practice eligible for linkage with the HES dataset were eligible for inclusion in the study. Patients with a diagnosis of CLD before the start of their follow-up period were excluded from the population. Patients were followed up starting at the latest of either the day after the date of current registration with their GP practice, the start of the study period or the date the GP practice was labelled "up to standard" (UTS). Follow-up ended at the earliest of either the date of death, date the patient transferred out of the GP practice, last date of data collection for the GP practice the patient is registered with, the end of the study period or the date of diagnosis with CLD in primary care (see Supplementary Table S1).

Outcomes

The frequency of testing recorded within the study period in general practice for the following liver blood tests was calculated: Alanine aminotransferase (ALT); Aspartate aminotransferase (AST); Gamma glutamyl transferase (GTT); Alkaline phosphatase (ALP); Bilirubin and platelet count. These markers were

For peer review only

selected as being routinely utilised in UK primary care for the assessment of liver function. Abnormal results for each test were defined as: ALT result >50 (IU/L); AST result >40 (IU/L); ALP result >130 (IU/L); GGT result >50 (IU/L); Bilirubin result >21 (IU/L); platelet result <150 (platelets/mcl).

Subgroups

Analyses were conducted in the following subgroups of patients at high risk of developing CLD: presence of type 2 diabetes defined using READ codes (see Supplementary Table S1); obesity defined as a BMI >30 calculated using height and weight measures; use of alcohol defined as excessive use of alcohol using READ codes (see Supplementary Table S1) or recorded >14 units per week alcohol consumption. For all subgroups, follow-up for an individual patient started at the date of diagnosis in primary care. Patients who were diagnosed with CLD within their follow up period had their follow-up shortened to end 3 months before their date of diagnosis with CLD. An analysis of the subgroup of patients not included in any of these high-risk subgroups was also performed.

Statistical Analysis

Characteristics of the population were compared using Chi-sq or Students t-test as appropriate to the data distribution. The frequency of liver blood testing was presented as the proportion of patients with one or more tests out of the total eligible population over the study period. The frequency of abnormal test results was calculated and presented as the proportion of non-missing test results with an abnormal value. The number of tests performed per year on an individual was calculated by dividing the number of tests performed in the individual's follow-up period divided by the total length of their follow-up period. The proportion of patients with an AST test within 6 weeks following an abnormal ALT test result was calculated.

All analyses were conducted overall and stratified by sex, age group (18 – 29, 30 – 39, 40 – 49, 50 – 59, 60 –69, 70 – 79, 80+ years) and calendar year. Analyses were performed on the whole study population and in the risk subgroups.

Analyses were performed using SAS version 9.4.

For peer review only

Patient and Public Involvement

This study involved members of the Nottingham Digestive Diseases Biomedical Research Unit Patient Advisory Group at the following stages: research design and funding application, lay dissemination and discussion of results.

For peer review only

Results

Characteristics

The study cohort included 2,912,066 individuals with follow-up during the years 2004 – 2016 (median follow-up 3.2 years, IQR 1.3-6.9). Of these, the predefined risk factor subgroups contained: 550,185 (19%) with obesity, 384,011 (13%) with excess alcohol use, 120,305 (4%) with type 2 diabetes and 2,235,938 (77%) with none of the three risk factors. 1480 individuals had all three risk factors.

The most frequently measured blood marker was platelet count, with 49% of patients having at least one platelet count measured during their follow-up period. The least commonly measured was the AST level with only 12% of patients having at least one measurement in their follow-up. For all tests, the prevalence of testing increased with increasing age, with the highest proportion of patients being tested in the 70 – 80 year age category. Markers were more frequently measured in women and this difference was statistically significant for all markers ($p<0.0001$). Full details are given in Table 1.

Of those participants having tests the median number of tests undertaken each year was 1, however some individuals had in excess of 100 of the same test per year. Platelet count was most likely to be tested more than once in an individual with the other liver markers being similar (for additional detail see Supplementary Table S2).

Prevalence of marker measurement over time

The proportion of patients in the study population with at least one measurement for ALT, ALP, Bilirubin or platelet test gradually increased over the course of the study period (2004 – 2016) but conversely fell for AST and GGT markers (Figure 1 and Table 2). By 2016 the proportion of the population receiving one or more of each test in that year was: platelet count 28.0%, ALP 26.2%, bilirubin 25.6%, ALT 23.7%, GGT 5.1% and AST 2.2%.

Prevalence of abnormal measures

The proportion of all tests being measured as abnormal remained generally static over the study period (Figure 2). Of the 3,922,529 (total number) of ALT test, 343,474 (8.8%) had an abnormal value. The first abnormal ALT test for each patient (N= 160,191) was paired with an AST test measurement within 6 weeks for 13,997 (8.7%). The proportion of measurements with abnormal values for all other markers

For peer review only

was also low: AST (7.5%), ALP (7.9%), GGT (24.6%), Bilirubin (4.7%), platelets (16.0%) and these proportions remained stable over the study period.

For peer review only

Table 1 Characteristics of participants (ever measurements)

		Whole population (N)	ALT		AST		ALP		GGT		Bilirubin		Platelet count	
			1+ tests	1+ abnormal	1+ tests	1+ abnormal	1+ tests	1+ abnormal	1+ tests	1+ abnormal	1+ tests	1+ abnormal	1+ tests	1+ abnormal
All	n	2,912,066	1,112,879	160,191	284,274	33743	1,261,596	140,433	459,754	124,475	1,246,003	99,633	1,414,798	301,127
	%		38.2%	14.4%	9.8%	11.9%	43.3%	11.1%	15.8%	27.1%	42.8%	8.0%	48.6%	21.3%
Sex														
Male	n	1,378,945	484,471	102,962	125,241	19,852	547,936	57,766	213,668	76,266	542,549	62,838	555,508	124,432
	%		35.1%	21.3%	9.1%	15.9%	39.7%	10.5%	15.5%	35.7%	39.3%	11.6%	40.3%	22.4%
Female	n	1,533,121	628,408	57,229	159,033	13,891	713,660	82,667	246,086	48,209	703,454	36,795	859,290	176,695
	%		41.0%	9.1%	10.4%	8.7%	46.5%	11.6%	16.1%	19.6%	45.9%	5.2%	56.0%	20.6%
Age group, years														
18 – 29	n	1,117,738	196,244	19,026	46,304	3,311	230,084	15,495	70,498	7,038	225,208	15,343	328,696	65,280
	%		17.6%	9.7%	4.1%	7.2%	20.6%	6.7%	6.3%	10.0%	20.1%	6.8%	29.4%	19.9%
30 – 39	n	1,000,314	246,015	35,067	61,081	6,127	287,277	18,522	95,123	19,394	281,707	18,460	370,007	74,137
	%		24.6%	14.3%	6.1%	10.0%	28.7%	6.4%	9.5%	20.4%	28.2%	6.6%	37.0%	20.0%
40 – 49	n	718,585	266,922	42,817	66,307	7,793	307,249	19,276	108,280	30,790	303,132	20,614	324,775	62,005
	%		37.1%	16.0%	9.2%	11.8%	42.8%	6.3%	15.1%	28.4%	42.2%	6.8%	45.2%	19.1%
50 - 59	n	476,113	221,016	36,113	54,770	72,51	251,719	24,052	91,799	30,896	248,874	16,947	247,761	47,522
	%		46.4%	16.3%	11.5%	13.2%	52.9%	9.6%	19.3%	33.7%	52.3%	6.8%	52.0%	19.2%
60 - 69	n	323,139	187,210	24,880	46,118	5,646	210,752	25,495	77,627	25,207	208,949	16,616	200,591	39,762
	%		57.9%	13.3%	14.3%	12.2%	65.2%	12.1%	24.0%	32.5%	64.7%	8.0%	62.1%	19.8%
70 - 79	n	212,186	133,592	11,776	33,557	3,637	150,577	23,758	54,137	16,123	149,271	13,196	145,700	31,741
	%		63.0%	8.8%	15.8%	10.8%	71.0%	15.8%	25.5%	29.8%	70.3%	8.8%	68.7%	21.8%
80+	n	182,665	106,411	6,586	25,860	2,436	120,156	28,719	41,325	11,671	118,880	9,989	123,721	28,849
	%		58.3%	6.2%	14.2%	9.4%	65.8%	23.9%	22.6%	28.2%	65.1%	8.4%	67.7%	23.3%

ALP alkaline phosphatase; **ALT** alanine aminotransferase; **AST** aspartate aminotransferase; **GGT** gamma glutamyl transferase

For peer review only

Table 2. Annual frequency of testing per patient in those with at least 1 test

		ALT	AST	GGT	ALP	Bilirubin	Platelet count
Median (IQR) maximum number of tests		1 (1-2) 108	1 (1-1) 47	1 (1-1) 45	1 (1-2) 131	1 (1-2) 108	1 (1-2) 92
1	n	1,914,577	436,400	691,189	2,129,817	2,200,429	1,972,278
	%	74.2%	75.5%	75.6%	70.2%	74.1%	60.1%
2	n	457,080	99,123	155,168	610,628	528,607	849,020
	%	17.7%	17.1%	17.0%	20.1%	17.8%	25.9%
3	n	121,616	24,862	40,197	164,364	139,762	185,136
	%	4.7%	4.3%	4.4%	5.4%	4.7%	5.6%
4	n	39,173	8,279	13,221	61,498	45,583	153,538
	%	1.5%	1.4%	1.4%	2.0%	1.5%	4.7%
5	n	15,569	3,336	4,960	23,572	18,166	29,952
	%	0.6%	0.6%	0.5%	0.8%	0.6%	0.9%
6-10	n	22,702	4,909	7,084	32,002	26,262	73,577
	%	0.9%	0.8%	0.8%	1.1%	0.9%	2.2%
11+	n	9,349	1,400	2,610	11,234	10,248	18,286
	%	0.4%	0.2%	0.3%	0.4%	0.3%	0.6%

ALP alkaline phosphatase; **ALT** alanine aminotransferase; **AST** aspartate aminotransferase; **GGT** gamma glutamyl transferase

For peer review only

Risk factor subgroup analyses

The prevalence of liver marker testing over time by the subgroups (no liver risk factors, excess alcohol consumption and/or obesity) showed similar trends to those for the whole population and are shown in Supplementary figure S1 and Supplementary table S3.

People with type 2 diabetes had a notably higher prevalance of testing for all markers (e.g. in 2016 ALT measured in 68.8% of those with type 2 diabetes vs 15.3% and 21.9% of those with alcohol excess and obesity respectively. However, the rates of decline in measurement of AST and GGT were also faster in those with diabetes than the other groups; for AST falling from 24.3% in 2004 to 6.5% in 2016 vs 6.5% and 9.8% to 3.0% and 3.4% of those with alcohol excess and obesity respectively; and for GGT falling from 28.6% in 2004 to 13.1% in 2016 vs 9.7% and 11.8% to 7.3% and 7.7% of those with alcohol excess and obesity respectively.

People with no risk factors for liver disease had the lowest prevalence of liver marker testing for all markers, however did still follow the same trends over time – increasing for ALT, ALP, bilirubin and platelets, and falling for AST and GGT.

For peer review only

Discussion

We found that whilst the majority of liver blood markers have shown increased rates of use in general practice over the past 10 years there was wide variation by both marker and subgroups of the population. Most notably, the use of AST has fallen to only 2% per annum amongst all general practice users.

The striking finding that AST is now only measured in a fraction of the population has significant implications for policy and practice. Major international guidelines, including American, European and British[7,17,18] all utilize non-invasive markers for investigating liver disease at a community level. AST is a critical component of Fib-4 which has been suggested as a first line test; to rule out significant disease. The absence of AST as a routinely collected marker presents a major barrier to the current implementation of pathways that attend to the aforementioned guidelines. This finding is consistent with other publications, where for example, in the assessment of liver fibrosis in individuals with a diagnosis of NAFLD only 11% had the necessary measures to allow the assessment of Fib-4 in the UK (rising to 54% in Catalonia, Spain)[19]. Furthermore, we found that <9% of abnormal ALT measurements also had an AST measured within a 6 week window.

The decision to prioritise ALT measurement over AST may have been driven by a push for efficiency savings[20] with ALT being considered more valuable as it is more liver specific. However AST may be a more sensitive indicator of chronic liver injury[21–23] especially when used as a ratio with ALT. In some regions an AST is automatically added if the ALT measure is abnormal to facilitate the AST/ALT ratio[24]. Over the 12year period examined nearly 40% of the population had at least one ALT measurement. This far exceeds the proportion of the known population dying prematurely of liver disease (estimated at 26,265 premature deaths in England in 2015-2017[25]), or the prevalence of recognised hepatic cirrhosis (estimated at 76.3 per 100,000 in 2001)[26]. Though the level of CLD in the UK is not known it is unlikely therefore that these tests are all done in those who have it or even are at high risk, and we therefore have to question why they are being performed and the opportunity cost it represents. Existing evidence suggests they are more often measured as part of routine monitoring than for CLD identification[27,28], and that discontinuation of such drugs rarely results[29]. If all these abnormalities were to be followed up (in accordance with existing guidance) there would be significant implications for downstream services. This includes the cost of a full liver screen, liver ultrasound and onward consultation and investigation in secondary care (e.g. national tariff for ultrasound scan £75.50, new

For peer review only

patient consultant led hepatology outpatient appointment is £208.56[30]). Furthermore, there is growing evidence that in advanced liver disease many individuals have a normal ALT[10,31], so the growth in use of this marker as a trigger for further assessment may still not identify liver disease.

A more nuanced approach where non-invasive markers are targeted towards individuals with risk factors for CLD may be one solution. From a diagnostic perspective it increases the pre-test probability of having disease and indeed this approach has been shown to be cost effective regardless of choice of biomarker[32,33] and region studied[34]. Within CPRD those patients with risk factors for CLD, as expected, had higher proportions receiving liver markers assessment than those without risk factors. However, this was still very varied by 2016, with 70% of individuals with T2DM having an ALT measure that year, more than double those with obesity and nearly three times those with alcohol excess – with all three groups having similar proportions of abnormal results. Whilst AST testing was more frequent amongst those with risk factors than in those without it was still very low (<8% in all groups). Therefore, from an implementation perspective it would make sense to focus efforts of obtaining AST and ALT in these groups, appreciating as step change in management is needed.

The strengths of this population approach are driven by the use of a dataset known to be broadly representative of the UK population in terms of age, gender and geographical location with robust quality controls[14] and also the use of validated code lists for subgroup identification[35]. It is therefore reasonable to assume that our findings regarding the level of testing overall and in subgroups are representative of what is happening in the UK. A key limitation is the lack of information on the indication for testing or the resultant actions which clearly limits interpretation to some degree. Additionally, since this study only includes people who attend the GP, some of the individuals at highest risk of CLD will not be attending, the estimates of the proportion of tests which would be abnormal with more systematic testing may be less accurate. A further issue is the lack of information to allow assessment of different liver blood testing systems, e.g. which areas 'package' different blood tests together or where abnormal results automatically trigger additional tests.

In conclusion, large numbers of liver blood markers are being measured annually in UK primary care. At present, they are not suitable for risk stratifying high risk populations for CLD as the key element (AST) required to calculate non-invasive fibrosis markers is missing. However, the highest risk groups are receiving regular blood testing (69%% of those with diabetes and 22% of those with obesity) so routine or opportunistic risk stratification could be feasible with limited additional expense to the NHS.

For peer review only

Funding

This study was funded as part of a Medical Research Council Clinician Scientist award held by JRM (grant number MR/P008348/1).

Conflict of interest

Nil to declare.

Authorship

The authors confirm contribution to the paper as follows: study conception and design: JRM, JW, TCC; data collection: JRM; analysis and interpretation of results: JRM, PS, LB, JW, TCC, CC; draft manuscript preparation: JRM, PS, ING. All authors reviewed the results and approved the final version of the manuscript.

Data sharing

This data was extracting following approval from the Clinical Practice Research Datalink (CPRD, https://www.cprd.com/). Applications for access should be directed directly to CPRD.

For peer review only

References

1. Public Health England. *Liver Disease: Applying All Our Health [Internet]*. https://www.gov.uk/government/publications/liver-disease-applying-all-our-health/liver-disease-applying-all-ourhealth accessed 10.03.2021 https://www.gov.uk/government/publications/liver-disease-applying-all-our-health/liver-disease-applying-all-ourhealth accessed 10.03.2021

2. Public Health England. Liver Disease Profiles [Internet]. Accessed February 23, 2016. http://fingertips.phe.org.uk/profile/liver-disease accessed 25 June 2020

3. Public Health England. *The 2nd Atlas of Variation in Risk Factors and Healthcare for Liver Disease in England. Reducing Unwarranted Variation to Improve Health Outcomes and Value [Internet]*. http://tools.england.nhs.uk/images/LiverAtlas17/atlas.html; 2017. https://www.gov.uk/government/publications/liver-disease-applying-all-our-health/liver-disease-applying-all-ourhealth accessed 10.03.2021

4. Williams R, Aspinall R, Bellis M, et al. Addressing liver disease in the UK: a blueprint for attaining excellence in health care and reducing premature mortality from lifestyle issues of excess consumption of alcohol, obesity, and viral hepatitis. *The Lancet*. 2014;384(9958):1953-1997. http://linkinghub.elsevier.com/retrieve/pii/S0140673614618389

5. Davies S. Chief Medical Officer annual report 2011: volume 1, chapter 2c, p 163. *Dep Health Novemb 2012 Available Httpswwwgovukgovernmentuploadssystemuploadsattachmentdatafile141773CMOAnnualReport2011Chapter2cpdf Accessed 01072015*. Published online 2012.

6. Liver Disease: Today's complacency, tomorrow's catastrophe. All-Party Parliamentary Hepatology Group (APPHG) Inquiry into Improving Outcomes in Liver Disease. *Available HttpwwwiasorgukuploadsAPPHG20report20March20201420FINALpdf Accessed 01072015*. Published online 2014.

7. Newsome PN, Cramb R, Davison SM, et al. Guidelines on the management of abnormal liver blood tests. *Gut*. 2018;67(1):6-19.

8. National Institute for Health and Care Excellence. NICE guidelines [NG49] Non-alcoholic fatty liver disease (NAFLD): assessment and management. Published online July 2016. Accessed September 8, 2016. https://www.nice.org.uk/guidance/ng49

9. National Institute for Health and Care Excellence. NICE guidelines [NG50] Cirrhosis in over 16s: assessment and management. Published online July 2016. Accessed September 8, 2016. https://www.nice.org.uk/guidance/ng50

10. Chalmers J, Wilkes E, Harris R, et al. Development and implementation of a commissioned pathway for the identification and stratification of liver disease in the community. *Frontline Gastroenterol*. Published online June 26, 2019:flgastro-2019-101177. doi:10.1136/flgastro-2019-101177

For peer review only

11. Wai CT, Greenson JK, Fontana RJ, et al. A simple noninvasive index can predict both significant fibrosis and cirrhosis in patients with chronic hepatitis C. *Hepatology*. 2003;38(2):518-526. http://dx.doi.org/10.1053/jhep.2003.50346

12. Vallet-Pichard A, Mallet V, Nalpas B, et al. FIB-4: An inexpensive and accurate marker of fibrosis in HCV infection. comparison with liver biopsy and fibrotest. *Hepatology*. 2007;46(1):32-36. doi:10.1002/hep.21669

13. Hydes T, Moore M, Stuart B, et al. Can routine blood tests be modelled to detect advanced liver disease in the community: model derivation and validation using UK primary and secondary care data. *BMJ Open*. 2021;11(2):e044952. doi:10.1136/bmjopen-2020-044952

14. Herrett E, Gallagher AM, Bhaskaran K, et al. Data Resource Profile: Clinical Practice Research Datalink (CPRD). *Int J Epidemiol*. 2015;44(3):827-836.

15. Herbert A, Wijlaars L, Zylbersztejn A, Cromwell D, Hardelid P. Data Resource Profile: Hospital Episode Statistics Admitted Patient Care (HES APC). *Int J Epidemiol*. 2017;46(4):1093-1093i. doi:10.1093/ije/dyx015

16. Padmanabhan S, Carty L, Cameron E, Ghosh RE, Williams R, Strongman H. Approach to record linkage of primary care data from Clinical Practice Research Datalink to other health-related patient data: overview and implications. *Eur J Epidemiol*. 2019;34(1):91-99. doi:10.1007/s10654-018-0442-4

17. Berzigotti A, Tsochatzis E, Boursier J, et al. EASL Clinical Practice Guidelines on non-invasive tests for evaluation of liver disease severity and prognosis – 2021 update. *J Hepatol*. Published online June 21, 2021. doi:10.1016/j.jhep.2021.05.025

18. Chalasani N, Younossi Z, Lavine JE, et al. The diagnosis and management of nonalcoholic fatty liver disease: Practice guidance from the American Association for the Study of Liver Diseases. *Hepatology*. 2018;67(1):328-357. doi:10.1002/hep.29367

19. Alexander M, Loomis AK, Fairburn-Beech J, et al. Real-world data reveal a diagnostic gap in non-alcoholic fatty liver disease. *BMC Med*. 2018;16(1):1-11.

20. Xu Q, Higgins T, Cembrowski GS. Limiting the Testing of AST: A Diagnostically Nonspecific Enzyme. *Am J Clin Pathol*. 2015;144(3):423-426. doi:10.1309/AJCPO47VAWYRIDHG

21. Daniel S, Ben-Menachem T, Vasudevan G, Ma CK, Blumenkehl M. Prospective evaluation of unexplained chronic liver transaminase abnormalities in asymptomatic and symptomatic patients. *Am J Gastroenterol*. 1999;94(10):3010-3014. doi:10.1016/S0002-9270(99)00507-9

22. Whitehead MW, Hawkes ND, Hainsworth I, Kingham JGC. A prospective study of the causes of notably raised aspartate aminotransferase of liver origin. *Gut*. 1999;45(1):129-133. doi:10.1136/gut.45.1.129

23. Lilford RJ, Bentham L, Girling A, et al. *Birmingham and Lambeth Liver Evaluation Testing Strategies (BALLETS): A Prospective Cohort Study*. NIHR Journals Library; 2013.

For peer review only

24. Dillon JF, Miller MH, Robinson EM, et al. Intelligent liver function testing (iLFT): A trial of automated diagnosis and staging of liver disease in primary care. *J Hepatol*. 2019;71(4):699-706.

25. Public Health Profiles. Accessed June 25, 2020. https://fingertips.phe.org.uk/profile/liver-disease

26. Fleming KM, Aithal GP, Solaymani-Dodaran M, Card TR, West J. Incidence and prevalence of cirrhosis in the United Kingdom, 1992–2001: a general population-based study. *J Hepatol*. 2008;49(5):732-738.

27. Standing HC, Jarvis H, Orr J, et al. GPs' experiences and perceptions of early detection of liver disease: a qualitative study in primary care. *Br J Gen Pr*. 2018;68(676):e743-e749.

28. Patel PJ, Banh X, Horsfall LU, et al. Underappreciation of non-alcoholic fatty liver disease by primary care clinicians: limited awareness of surrogate markers of fibrosis. *Intern Med J*. 2018;48(2):144-151.

29. Nakafero G, Grainge MJ, Card T, et al. What is the incidence of methotrexate or leflunomide discontinuation related to cytopenia, liver enzyme elevation or kidney function decline? *Rheumatology*. 2021;(keab254). doi:10.1093/rheumatology/keab254

30. UK Department of Health. National Cost Collection: National Schedule of NHS costs - Year 2019-20 - NHS trust and NHS foundation trusts. Availble at https://www.england.nhs.uk/national-cost-collection/.

31. Fracanzani AL, Valenti L, Bugianesi E, et al. Risk of severe liver disease in nonalcoholic fatty liver disease with normal aminotransferase levels: A role for insulin resistance and diabetes. *Hepatology*. 2008;48(3):792-798. doi:https://doi.org/10.1002/hep.22429

32. Srivastava A, Jong S, Gola A, et al. Cost-comparison analysis of FIB-4, ELF and fibroscan in community pathways for non-alcoholic fatty liver disease. *BMC Gastroenterol*. 2019;19(1):122. doi:10.1186/s12876-019-1039-4

33. Tanajewski L, Harris R, Harman DJ, et al. Economic evaluation of a community-based diagnostic pathway to stratify adults for non-alcoholic fatty liver disease: a Markov model informed by a feasibility study. *BMJ Open*. 2017;7(6):e015659. doi:10.1136/bmjopen-2016-015659

34. Serra-Burriel M, Graupera I, Torán P, et al. Transient elastography for screening of liver fibrosis: cost-effectiveness analysis from six prospective cohorts in Europe and Asia. *J Hepatol*. 2019;71(6):1141-1151.

35. Ratib S, West J, Crooks CJ, Fleming KM. Diagnosis of Liver Cirrhosis in England, a Cohort Study, 1998-2009: A Comparison With Cancer. *Am J Gastroenterol*. 2014;109(2):190-198. http://dx.doi.org/10.1038/ajg.2013.405

For peer review only

Figure 1 Prevalence of liver enzyme testing amongst adults over time

ALP alkaline phosphatase; **ALT** alanine aminotransferase; **AST** aspartate aminotransferase;

GGT gamma glutamyl transferase

Figure 2 Prevalence of abnormal values of liver blood tests in adults over time

ALP alkaline phosphatase; **ALT** alanine aminotransferase; **AST** aspartate aminotransferase;

GGT gamma glutamyl transferase

For peer review only

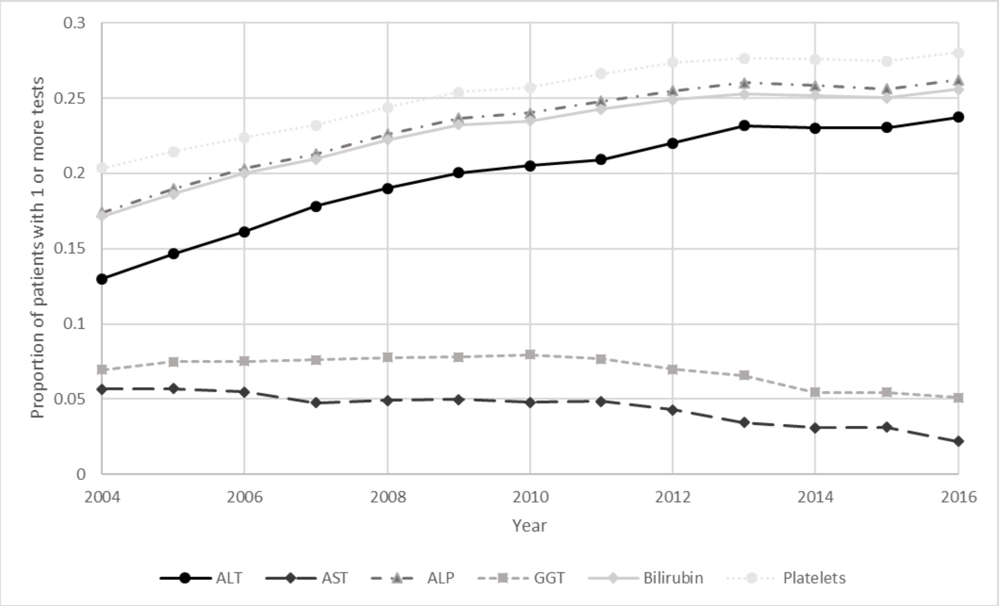

Figure 1 Prevalence of liver enzyme testing amongst adults over time

ALP alkaline phosphatase; ALT alanine aminotransferase; AST aspartate aminotransferase; GGT gamma glutamyl transferase

456x277mm (59 x 59 DPI)

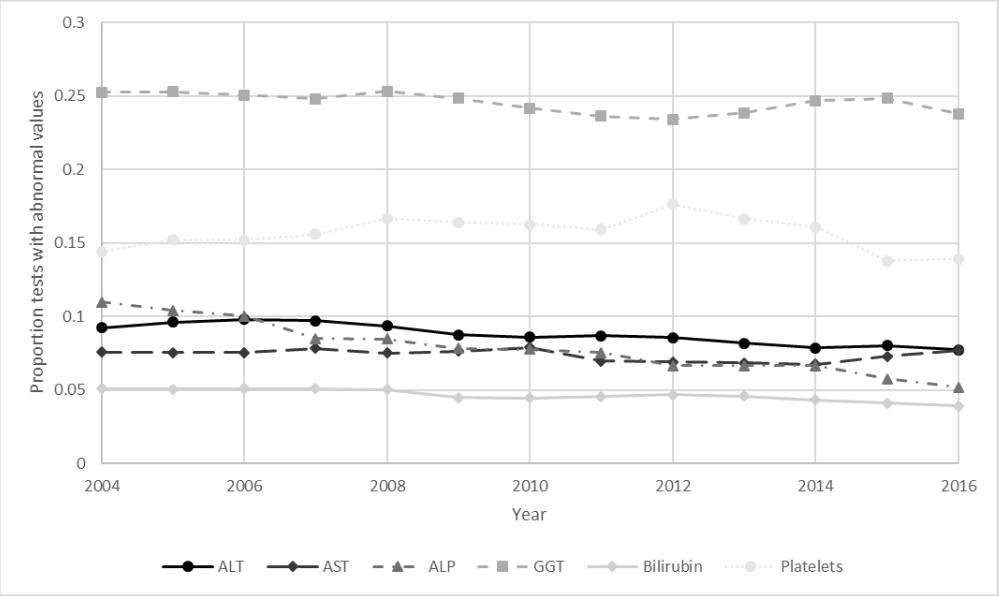

Figure 2 Prevalence of abnormal values of liver blood tests in adults over time

ALP alkaline phosphatase; ALT alanine aminotransferase; AST aspartate aminotransferase; GGT gamma glutamyl transferase

459x274mm (59 x 59 DPI)

Supplementary Table S1 – Codes

Liver blood tests and their values were identified from the CPRD "test" dataset using the following entity file codes: ALT = 155; AST = 156; ALP = 153; GGT = 172; Bilirubin = 158; Platelet count = 189.

Type 2 diabetes

C100112	Non-insulin dependent diabetes mellitus	C109200	Non-insulin-dependent diabetes mellitus with neuro comps
C107400	NIDDM with peripheral circulatory disorder	C109211	Type II diabetes mellitus with neurological complications
C109.00	Non-insulin dependent diabetes mellitus	C109212	Type 2 diabetes mellitus with neurological complications
C109.11	NIDDM - Non-insulin dependent diabetes mellitus	C109300	Non-insulin-dependent diabetes mellitus with multiple comps
C109.12	Type 2 diabetes mellitus	C109312	Type 2 diabetes mellitus with multiple complications
C109.13	Type II diabetes mellitus	C109400	Non-insulin dependent diabetes mellitus with ulcer
C109000	Non-insulin-dependent diabetes mellitus with renal comps	C109411	Type II diabetes mellitus with ulcer
C109011	Type II diabetes mellitus with renal complications	C109412	Type 2 diabetes mellitus with ulcer
C109012	Type 2 diabetes mellitus with renal complications	C109500	Non-insulin dependent diabetes mellitus with gangrene
C109100	Non-insulin-dependent diabetes mellitus with ophthalm comps	C109511	Type II diabetes mellitus with gangrene
C109111	Type II diabetes mellitus with ophthalmic complications	C109512	Type 2 diabetes mellitus with gangrene
C109112	Type 2 diabetes mellitus with ophthalmic complications	C109600	Non-insulin-dependent diabetes mellitus with retinopathy

Obesity = BMI>30. Values obtained from entity file: weight =13 and height = 14.
Calculate BMI for every available weight measurement (using single height measurement). If BMI > 30 at any stage up to end of follow-up for individual patient then include in Obesity population. Index date is first occurrence of BMI >30.

Cirrhosis: Identified from READ codes/terms

7609	Open operations on oesophageal varices	J612.12	Laennec's cirrhosis
7609300	Local ligation of oesophageal varices	J615.00	Cirrhosis - non alcoholic
7609400	Open injection sclerotherapy to oesophageal varices	J615.11	Portal cirrhosis
7609y11	Tanner devascularisation for bleeding varices	J615100	Multilobular portal cirrhosis
7609z00	Open operation on oesophageal varices NOS	J615300	Diffuse nodular cirrhosis
760C300	Fibreoptic endoscopic injection sclerotherapy oesoph varices	J615400	Fatty portal cirrhosis
760C500	Fibreoptic endoscopic banding of oesophageal varices	J615500	Hypertrophic portal cirrhosis
760F300	Rigid oesophagoscopic injection sclerotherapy oesoph varices	J615600	Capsular portal cirrhosis
760F400	Rigid oesophagoscopic banding of oesophageal varices	J615700	Cardiac portal cirrhosis
761D800	Fibreopt endoscop rubber band ligation of upper GIT varices	J615800	Juvenile portal cirrhosis
C310400	Glycogenosis with hepatic cirrhosis	J615812	Indian childhood cirrhosis

For peer review only

C350012	Pigmentary cirrhosis of liver	J615C00	Xanthomatous portal cirrhosis
G85..11	Oesophageal varices	J615D00	Bacterial portal cirrhosis
G850.00	Oesophageal varices with bleeding	J615H00	Infectious cirrhosis NOS
G851.00	Oesophageal varices without bleeding	J615y00	Portal cirrhosis unspecified
G852.00	Oesophageal varices in diseases EC	J615z00	Non-alcoholic cirrhosis NOS
G852000	Oesophageal varices with bleeding in diseases EC	J615z11	Macronodular cirrhosis of liver
G852100	Oesophageal varices without bleeding in diseases EC	J615z12	Cryptogenic cirrhosis of liver
G852200	Oesophageal varices in cirrhosis of the liver	J615z13	Cirrhosis of liver NOS
G852300	Oesophageal varices in alcoholic cirrhosis of the liver	J616.00	Biliary cirrhosis
G852z00	Oesophageal varices in diseases EC NOS	J616100	Secondary biliary cirrhosis
G857.00	Gastric varices	J616200	Biliary cirrhosis of children
G858.00	Oesophageal varices NOS	J616z00	Biliary cirrhosis NOS
J61..00	Cirrhosis and chronic liver disease	J623.00	Portal hypertension
J612.00	Alcoholic cirrhosis of liver	J635600	Toxic liver disease with fibrosis and cirrhosis of liver
J612.11	Florid cirrhosis	Jyu7100	[X]Other and unspecified cirrhosis of liver
**Alcohol excess:** Identified from units per week from entity file (entity=5) and READ codes/terms			
136K.00	Alcohol intake above recommended sensible limits	E231.00	Chronic alcoholism
136S.00	Hazardous alcohol use	E231z00	Chronic alcoholism NOS
136T.00	Harmful alcohol use	E23z.00	Alcohol dependence syndrome NOS
136W.00	Alcohol misuse	E250.00	Nondependent alcohol abuse
8H7p.00	Referral to community alcohol team	E250000	Nondependent alcohol abuse, unspecified
9NN2.00	Under care of community alcohol team	E250200	Nondependent alcohol abuse, episodic
9k1..00	Alcohol misuse - enhanced services administration	E250z00	Nondependent alcohol abuse NOS
9k1A.00	Brief intervention for excessive alcohol consumption completed	Eu10211	[X]Alcohol addiction
E23..00	Alcohol dependence syndrome	Eu10212	[X]Chronic alcoholism
E23..11	Alcoholism	ZV11300	[V]Personal history of alcoholism
E23..12	Alcohol problem drinking		

For peer review only

Supplementary Table S2 – Total numbers of tests measured and individuals being tested

	2004	2005	2006	2007	2008	2009	2010	2011	2012	2013	2014	2015	2016
**ALT**													
Total tests	145,762	182,250	220,863	264,573	299,956	336,059	349,857	355,185	377,633	379,820	336,022	274,827	199,196
Total people tested	101,485	126,562	152,516	183,196	206,761	228,380	239,920	246,920	261,544	264,206	233,037	193,654	141,885
**AST**													
Total tests	62,414	69689	72,998	69218	75,502	81218	78,629	79997	71,305	53,714	44,064	36,542	18,363
Total people tested	44,294	49158	51,781	48791	53,406	56756	56,085	57243	50,856	39,178	31,411	26,275	13,075
**ALP**													
Total tests	199,350	241693	288,224	326253	368,076	411994	426,350	442311	461,329	454,319	405,753	332,655	236,886
Total people tested	136,121	163894	192,150	218956	245,608	269713	280,957	292771	302,822	296,931	261,378	215,265	156,549
**GGT**													
Total tests	78,572	92452	102,634	111127	119,522	127386	132,905	127210	116,707	102,085	74,190	61,516	40,953
Total people tested	54,346	64692	71,192	78118	84,354	88723	93,147	90618	82,859	75,125	55,022	45,726	30,507
**Bilirubin**													
Total tests	192,632	231683	275,631	311066	350,373	388612	399,189	411125	426,237	412,887	366,861	299,687	215,528
Total people tested	134,046	160842	189,233	215296	241,713	264757	274,782	286590	295,722	288,212	254,867	210,081	152,916
**Platelet count**													
Total tests	269,019	318699	368,616	419187	475,732	524655	540,431	555732	589,239	557,555	495,158	389,745	279,714
Total people tested	158,987	185336	211,828	238400	265,257	289243	300,868	314311	324,861	315,432	279,168	230,590	167,506

For peer review only

Supplementary Table S3 Prevalence of liver blood markers over time

	2004	2005	2006	2007	2008	2009	2010	2011	2012	2013	2014	2015	2016
**ALT**													
All	13.0% 101,485	14.7% 126,562	16.1% 152,516	17.8% 183,196	19.0% 206,761	20.0% 228,380	20.5% 239,920	20.9% 246,920	22.0% 261,544	23.2% 264,206	23.0% 233,037	23.1% 193,654	23.7% 141,885
No risk factor	10.2% 58,099	11.5% 71,085	12.3% 82,630	13.7% 97,925	14.6% 109,206	15.4% 119,415	15.6% 122,910	15.9% 125,077	16.7% 131,327	17.5% 131,491	17.4% 115,211	17.3% 93,323	17.9% 69,323
Alcohol excess	15.3% 15,571	17.2% 19,838	19.2% 24,616	21.5% 30,304	23.1% 34,840	24.4% 38,992	25.1% 41,558	25.6% 42,907	27.1% 45,955	28.3% 46,207	28.0% 41,346	27.4% 34,669	28.3% 23,270
Obesity	21.9% 25,460	24.6% 33,357	27.2% 42,787	29.2% 53,140	30.6% 61,862	31.6% 70,159	32.4% 76,530	32.5% 80,517	33.9% 86,275	35.4% 88,493	35.0% 79,136	35.1% 68,224	35.7% 50,029
Type 2 diabetes	56.1% 10,271	61.0% 13,741	64.4% 17,680	67.9% 21,964	68.4% 24,670	68.1% 27,140	68.9% 29,564	67.4% 31,100	68.5% 33,335	70.7% 34,909	68.4% 31,408	68.6% 27,066	68.8% 20,317
All 3 risk factors	59.1% 874	63.9% 1247	68.4% 1685	71.7% 2186	72.4% 2613	71.9% 3035	71.5% 3430	70.7% 3769	70.3% 3983	73.0% 4260	71.3% 3931	70.2% 3510	71.1% 2436
**AST**													
All	5.7% 44,294	5.7% 49,158	5.5% 51,781	4.7% 48,791	4.9% 53,406	5.0% 56,756	4.8% 56,085	4.8% 57,243	4.3% 50,856	3.4% 39,178	3.1% 31,411	3.1% 26,275	2.2% 13,075
No risk factor	4.4% 25,237	4.5% 27,674	4.1% 27,556	3.6% 25,603	3.7% 27,614	3.7% 28,920	3.6% 28,504	3.7% 29,130	3.3% 25,902	2.6% 19,882	2.3% 15,406	2.3% 12,526	1.6% 6,017
Alcohol excess	6.5% 6,672	6.5% 7,446	6.5% 8,311	5.5% 7,708	5.6% 8,497	5.7% 9,129	5.5% 9,123	5.5% 9,214	4.8% 8,206	3.9% 6,298	3.6% 5,368	3.8% 4,791	3.0% 2,503
Obesity	9.8% 11,411	9.7% 13,137	9.6% 15,047	8.2% 14,942	8.4% 17,073	8.3% 18,511	7.8% 18,354	7.6% 18,929	6.6% 16,709	5.3% 13,124	4.8% 10,921	4.8% 9,327	3.4% 4,740
Type 2 diabetes	24.3% 4,444	23.6% 5,309	22.8% 6,257	18.9% 6,104	18.9% 6,808	18.4% 7,318	16.8% 7,228	16.0% 7,387	13.6% 6,599	10.3% 5,099	9.1% 4,155	9.6% 3,780	6.5% 1,919
All 3 risk factors	24.9% 369	24.4% 477	22.6% 557	18.9% 577	18.7% 676	18.0% 759	16.5% 793	15.3% 813	12.7% 721	9.9% 576	9.2% 506	9.6% 478	7.4% 252
**ALP**													

For peer review only

All	17.4%	19.0%	20.3%	21.3%	22.6%	23.7%	24.0%	24.8%	25.5%	26.0%	25.8%	25.6%	26.2%
	136,121	163,894	192,150	218,956	245,608	269,713	280,957	292,771	302,822	296,931	261,378	215,265	156,549
No risk	13.8%	14.9%	15.6%	16.4%	17.4%	18.2%	18.3%	19.0%	19.5%	19.9%	19.7%	19.4%	19.9%
factor	78,382	92,690	104,627	117,407	129,995	141,235	144,684	149,282	153,272	149,168	130,327	104,727	76,926
Alcohol	20.0%	21.7%	23.8%	25.0%	26.6%	27.9%	28.6%	29.4%	30.5%	31.0%	30.7%	29.9%	31.1%
excess	20,355	25,039	30,447	35,275	40,123	44,632	47,212	49,230	51,624	50,602	45,304	37,836	25,565
Obesity	29.5%	31.9%	34.3%	35.1%	36.8%	37.7%	38.1%	38.7%	39.2%	39.7%	39.1%	38.8%	39.2%
	34,314	43,222	53,982	63,964	74,331	83,756	89,904	95,755	99,898	99,094	88,395	75,373	54,922
Type 2	73.5%	77.4%	79.6%	80.4%	81.0%	80.6%	80.2%	79.3%	78.6%	78.2%	75.5%	75.1%	75.0%
diabetes	13,458	17,429	21,845	26,018	29,210	32,115	34,420	36,568	38,245	38,590	34,647	29,619	22,161
All 3 risk	75.0%	79.1%	82.1%	82.0%	82.7%	81.9%	80.8%	80.8%	78.9%	78.7%	77.1%	75.5%	77.2%
factors	1,110	1,544	2,021	2,501	2,986	3,455	3,876	4,305	4,471	4,588	4,251	3,774	2,645
**GGT**													
All	7.0%	7.5%	7.5%	7.6%	7.8%	7.8%	8.0%	7.7%	7.0%	6.6%	5.4%	5.4%	5.1%
	54,346	64,692	71,192	78,118	84,354	88,723	93,147	90,618	82,859	75,125	55,022	45,726	30,507
No risk	5.3%	5.6%	5.5%	5.5%	5.6%	5.6%	5.7%	5.5%	5.0%	4.7%	3.9%	3.9%	3.7%
factor	29,868	34,865	36,661	39,595	42,214	43,632	45,103	43,616	39,378	35,625	25,434	20,846	14,408
Alcohol	9.7%	10.3%	10.8%	11.0%	11.3%	11.5%	11.7%	11.2%	10.4%	9.6%	8.3%	7.9%	7.3%
excess	9,837	11,909	13,845	15,572	17,021	18,307	19,323	18,790	17,571	15,738	12,271	9,922	5,971
Obesity	11.8%	12.8%	12.9%	12.6%	12.8%	12.6%	12.8%	12.1%	10.9%	10.2%	8.4%	8.5%	7.7%
	13,754	17,296	20,350	23,024	25,877	27,954	30,300	29,929	27,680	25,385	19,030	16,469	10,731
Type 2	28.6%	29.3%	28.0%	27.1%	26.2%	25.1%	25.3%	23.7%	20.7%	19.2%	14.8%	15.1%	13.1%
diabetes	5,232	6,601	7,693	8,757	9,456	10,007	10,855	10,946	10,061	9,484	6,796	5,942	3,879
All 3 risk	32.2%	33.8%	33.0%	32.2%	31.3%	31.1%	30.1%	29.4%	24.6%	22.6%	18.3%	17.8%	14.8%
factors	476	659	812	982	1,131	1,314	1,443	1,568	1,394	1,319	1,009	891	507
**Bilirubin**													
All	17.2%	18.6%	20.0%	20.9%	22.2%	23.2%	23.5%	24.3%	24.9%	25.3%	25.2%	25.0%	25.6%
	134,046	160,842	189,233	215,296	241,713	264,757	274,782	286,590	295,722	288,212	254,867	210,081	152,916
	13.5%	14.6%	15.3%	16.1%	17.0%	17.8%	17.8%	18.4%	18.9%	19.2%	19.1%	18.8%	19.3%

For peer review only

No risk factor	76,925	90,589	102,695	114,978	127,452	137,983	140,694	145,318	148,912	143,914	126,200	101,520	74,600
Alcohol excess	19.8% 20,174	21.5% 24,766	23.5% 30,144	24.7% 34,890	26.4% 39,753	27.6% 44,116	28.2% 46,549	29.0% 48,609	30.0% 50,790	30.3% 49,536	30.2% 44,537	29.4% 37,217	30.7% 25,195
Obesity	29.1% 33,896	31.4% 42,574	33.9% 53,306	34.7% 63,125	36.3% 73,377	37.2% 82,584	37.4% 88,397	38.0% 94,097	38.5% 97,916	38.7% 96,601	38.3% 86,706	38.0% 73,942	38.5% 53,945
Type 2 diabetes	73.2% 13,402	76.9% 17,306	79.1% 21,709	79.9% 25,850	80.5% 29,030	80.0% 31,878	79.5% 34,108	78.7% 36,294	77.7% 37,812	77.0% 38,009	74.8% 34,346	74.5% 29,378	74.4% 21,978
All 3 risk factors	74.8% 1,107	78.7% 1,536	81.6% 2,010	81.8% 2,494	82.4% 2,976	81.6% 3,443	80.5% 3,862	80.4% 4,284	78.2% 4,430	77.9% 4,544	76.6% 4,222	75.0% 3,749	76.8% 2,629
**Platelets**													
All	20.3% 158,987	21.5% 185,336	22.4% 211,828	23.2% 238,400	24.4% 265,257	25.4% 289,243	25.7% 300,868	26.6% 314,311	27.4% 324,861	27.6% 315,432	27.6% 279,168	27.5% 230,590	28.0% 167,506
No risk factor	18.3% 104,008	19.1% 118,472	19.5% 130,674	20.1% 144,262	21.0% 157,524	21.8% 168,847	21.8% 172,317	22.5% 177,447	23.0% 180,888	23.1% 173,265	22.9% 151,378	22.4% 121,000	22.9% 88,638
Alcohol excess	18.6% 18,994	19.9% 22,933	21.4% 27,424	22.5% 31,684	24.0% 36,207	25.2% 40,327	26.0% 42,991	27.0% 45,170	28.4% 48,143	29.0% 47,396	29.1% 42,957	29.0% 36,654	30.0% 24,657
Obesity	28.7% 33,393	30.6% 41,452	32.4% 50,954	33.1% 60,244	34.7% 70,172	35.9% 79,767	36.5% 86,087	37.5% 92,765	38.3% 97,383	38.6% 96,496	38.6% 87,227	38.9% 75,562	39.4% 55,216
Type 2 diabetes	47.9% 8,764	51.1% 11,511	54.4% 14,932	56.1% 18,153	58.3% 21,031	59.5% 23,689	61.9% 26,553	63.2% 29,146	64.0% 31,107	64.4% 31,793	63.7% 29,233	65.3% 25,752	66.3% 19,593
All 3 risk factors	43.9% 650	48.6% 949	51.9% 1,278	52.9% 1,614	56.0% 2,020	56.9% 2,403	59.1% 2,835	61.2% 3,261	61.4% 3,477	63.0% 3,674	62.7% 3,455	64.2% 3,210	67.8% 2,323

For peer review only

Supplementary Figure S1 Prevalence of liver marker testing over time by the subgroups: no liver risk factors, Type 2 diabetes, excess alcohol consumption and obesity

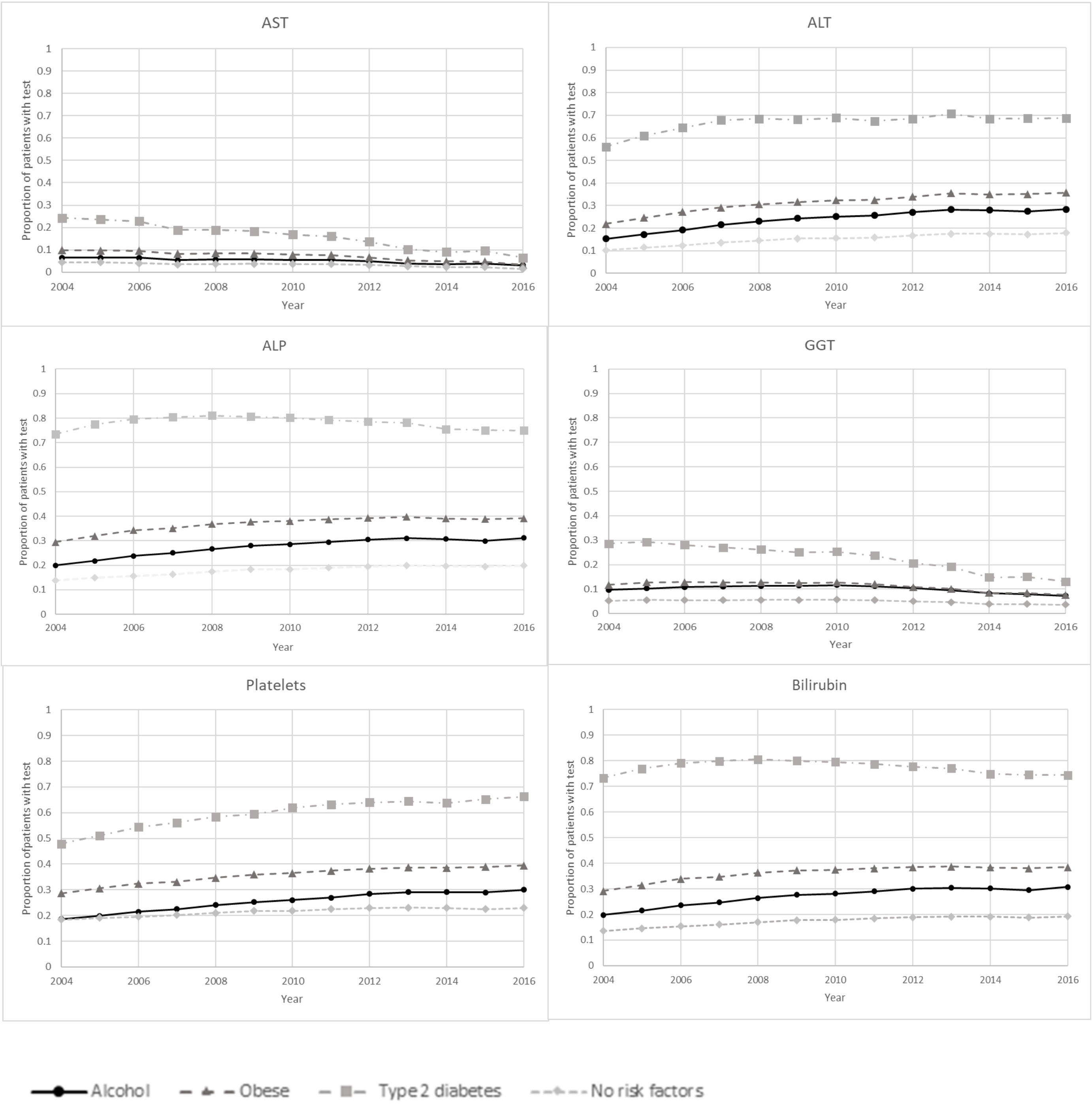

STROBE Statement—checklist of items that should be included in reports of observational studies

	Item No	Recommendation	
**Title and abstract**	1	(*a*) Indicate the study's design with a commonly used term in the title or the abstract	1
		(*b*) Provide in the abstract an informative and balanced summary of what was done and what was found	2
**Introduction**			
Background/rationale	2	Explain the scientific background and rationale for the investigation being reported	4
Objectives	3	State specific objectives, including any prespecified hypotheses	4
**Methods**			
Study design	4	Present key elements of study design early in the paper	5
Setting	5	Describe the setting, locations, and relevant dates, including periods of recruitment, exposure, follow-up, and data collection	5
Participants	6	(*a*) *Cohort study*—Give the eligibility criteria, and the sources and methods of selection of participants. Describe methods of follow-up<br>*Case-control study*—Give the eligibility criteria, and the sources and methods of case ascertainment and control selection. Give the rationale for the choice of cases and controls<br>*Cross-sectional study*—Give the eligibility criteria, and the sources and methods of selection of participants	5
		(*b*) *Cohort study*—For matched studies, give matching criteria and number of exposed and unexposed<br>*Case-control study*—For matched studies, give matching criteria and the number of controls per case	NA
Variables	7	Clearly define all outcomes, exposures, predictors, potential confounders, and effect modifiers. Give diagnostic criteria, if applicable	5/6
Data sources/ measurement	8*	For each variable of interest, give sources of data and details of methods of assessment (measurement). Describe comparability of assessment methods if there is more than one group	5/6
Bias	9	Describe any efforts to address potential sources of bias	5/6
Study size	10	Explain how the study size was arrived at	5
Quantitative variables	11	Explain how quantitative variables were handled in the analyses. If applicable, describe which groupings were chosen and why	6
Statistical methods	12	(*a*) Describe all statistical methods, including those used to control for confounding	6
		(*b*) Describe any methods used to examine subgroups and interactions	6
		(*c*) Explain how missing data were addressed	
		(*d*) *Cohort study*—If applicable, explain how loss to follow-up was addressed<br>*Case-control study*—If applicable, explain how matching of cases and controls was addressed<br>*Cross-sectional study*—If applicable, describe analytical methods taking account of sampling strategy	NA
		(*e*) Describe any sensitivity analyses	6

Continued on next page

For peer review only

**Results**			
Participants	13*	(a) Report numbers of individuals at each stage of study—eg numbers potentially eligible, examined for eligibility, confirmed eligible, included in the study, completing follow-up, and analysed	7
		(b) Give reasons for non-participation at each stage	7
		(c) Consider use of a flow diagram	NA
Descriptive data	14*	(a) Give characteristics of study participants (eg demographic, clinical, social) and information on exposures and potential confounders	7
		(b) Indicate number of participants with missing data for each variable of interest	7
		(c) *Cohort study*—Summarise follow-up time (eg, average and total amount)	7
Outcome data	15*	*Cohort study*—Report numbers of outcome events or summary measures over time	7/8
		Case-control study—Report numbers in each exposure category, or summary measures of exposure	
		Cross-sectional study—Report numbers of outcome events or summary measures	
Main results	16	(*a*) Give unadjusted estimates and, if applicable, confounder-adjusted estimates and their precision (eg, 95% confidence interval). Make clear which confounders were adjusted for and why they were included	7/8
		(*b*) Report category boundaries when continuous variables were categorized	7/8
		(*c*) If relevant, consider translating estimates of relative risk into absolute risk for a meaningful time period	
Other analyses	17	Report other analyses done—eg analyses of subgroups and interactions, and sensitivity analyses	8
**Discussion**			
Key results	18	Summarise key results with reference to study objectives	9
Limitations	19	Discuss limitations of the study, taking into account sources of potential bias or imprecision. Discuss both direction and magnitude of any potential bias	10
Interpretation	20	Give a cautious overall interpretation of results considering objectives, limitations, multiplicity of analyses, results from similar studies, and other relevant evidence	9/10
Generalisability	21	Discuss the generalisability (external validity) of the study results	10
**Other information**			
Funding	22	Give the source of funding and the role of the funders for the present study and, if applicable, for the original study on which the present article is based	10

*Give information separately for cases and controls in case-control studies and, if applicable, for exposed and unexposed groups in cohort and cross-sectional studies.

Note: An Explanation and Elaboration article discusses each checklist item and gives methodological background and published examples of transparent reporting. The STROBE checklist is best used in conjunction with this article (freely available on the Web sites of PLoS Medicine at http://www.plosmedicine.org/, Annals of Internal Medicine at http://www.annals.org/, and Epidemiology at http://www.epidem.com/). Information on the STROBE Initiative is available at www.strobe-statement.org.

For peer review only

For peer review only - http://bmjopen.bmj.com/site/about/guidelines.xhtml
